# HepDecide: A Standards-Oriented, Explainable Clinical Decision Support Prototype for the Chronic Liver-Disease Spectrum—Design, Guideline-Grounded Reporting, and a Leakage-Controlled Retrospective Evaluation

**DOI:** 10.3390/healthcare14203397

**Published:** 2026-10-12

**Authors:** Nazlı Tokatlı

**Affiliations:** Department of Computer Engineering, Faculty of Engineering and Natural Sciences, Istanbul Health and Technology University, 34445 Istanbul, Turkey; nazli.tokatli@istun.edu.tr

**Keywords:** clinical decision support systems, health informatics, digital health, HL7 FHIR, interoperability, explainable artificial intelligence, large language models, retrieval-augmented generation, retrospective evaluation, calibration, chronic liver disease

## Abstract

**Background/Objectives:** Specialist hepatology capacity is small relative to the population needing assessment, so most decisions about whom to investigate for chronic liver disease fall to non-specialists guided by static laboratory indices reported in isolation. This study presents HepDecide, a standards-oriented decision support prototype consolidating stage-specific risk estimation, explainability and guideline-traceable reporting into one physician-facing service, and reports a leakage-controlled retrospective evaluation of its predictive layer. **Methods:** HepDecide combines stage-specific tabular classifiers, an explainability layer based on SHapley Additive exPlanations (SHAP), and retrieval-augmented generation (RAG) anchored to EASL and AASLD guidelines; outputs are expressed as HL7 FHIR R4 resources, with inputs mapped to LOINC and diseases to ICD-10. Three endpoints were modelled on two publicly available, de-identified cohorts: steatohepatitis versus simple steatosis and histological cirrhosis (F4) in a biopsy-characterised MASLD cohort (*n*= 605), and one-year survival among patients with hepatocellular carcinoma (HCC; *n* = 165). Because patient-level predictions from the original development runs were not retained, the primary evaluation is a separate re-analysis with a locked patient-wise test partition, imputation, scaling and resampling fitted within training folds only, 25 classifier–imbalance configurations per endpoint, training-only calibration, and calibration, precision–recall, decision-curve and SHAP analyses. **Results:** On the locked test partitions, ROC-AUC was 0.67 (95% CI 0.50–0.84) for steatohepatitis (*n* = 121), 0.81 (0.52–1.00) for cirrhosis (*n* = 121; five events) and 0.76 (0.56–0.92) for one-year HCC survival (*n* = 33), with Brier scores of 0.096, 0.028 and 0.199. On the same cirrhosis test partition FIB-4 reached an ROC-AUC of 0.92 (0.82–0.99). The five highest-ranked SHAP features accounted for 58–73% of total attribution. Development-phase estimates of 0.90, 0.98 and 0.92 are reported separately as archived values; the cirrhosis estimate is documented in a retained training output, although its original input tables and individual test probabilities are unavailable. No quantitative evaluation of the language-generation layer is claimed, because the corresponding records were not retained. **Conclusions:** Under leakage-controlled retrospective conditions the predictive layer showed moderate discrimination with wide confidence intervals and did not outperform FIB-4 for cirrhosis. The value of the prototype lies in its standards-oriented, explainable and guideline-grounded design and in a fully documented, reproducible evaluation pipeline; transportability, the text-generation layer and clinical usefulness remain to be established prospectively. HepDecide is intended as a clinician-supervised triage and reporting prototype, not a clinically ready system.

## 1. Introduction

Metabolic dysfunction-associated steatotic liver disease (MASLD; formerly non-alcoholic fatty liver disease and MAFLD) has become the most prevalent chronic liver condition worldwide [1,2], and its downstream complications, cirrhosis and hepatocellular carcinoma (HCC), impose a growing and measurable burden on health systems [1,2,3]. The scale of that burden is a health-services problem before it is a modelling problem. The population at risk is far larger than specialist hepatology capacity can assess individually, so the decision of whom to investigate further is taken predominantly in primary and secondary care. It falls to clinicians who are not liver specialists and who have minutes rather than hours per patient. A clinically important subset of patients progresses along a pathological spectrum from simple steatosis through advanced fibrosis and cirrhosis to HCC, and population-based studies have documented incident cirrhosis and HCC even among patients without pre-existing cirrhosis [4]. Because early-stage disease is frequently asymptomatic while advanced stages are associated with irreversible morbidity and significant mortality, current European Association for the Study of the Liver (EASL) and American Association for the Study of Liver Diseases (AASLD) guidelines emphasise early risk identification and continuous monitoring [5,6], and individualised risk prediction in non-cirrhotic MASLD remains an active and unresolved clinical priority [7].

In routine clinical care, risk is assessed primarily through static, laboratory-based indices: fibrosis markers such as the fibrosis-4 index (FIB-4) and the aspartate aminotransferase-to-platelet ratio index (APRI) [8,9], and severity or prognostic scores such as the Model for End-Stage Liver Disease (MELD) score [10] and the Child–Pugh classification [11]. Although inexpensive and interpretable, these rule-based tools rely on fixed thresholds and a limited number of variables, rendering them unable to capture the non-linear, high-dimensional interactions among biomarkers. Furthermore, their fragmented presentation across clinical workflows increases clinician cognitive load and produces indeterminate ranges that reduce diagnostic sensitivity near decision boundaries. In direct comparisons, agreement between these scores is imperfect and no single index has been shown to perform adequately in every clinical setting in which it is used [12]. A critical gap thus remains between the numerical data available in the electronic health record (EHR) and the actionable clinical knowledge required at the point of care. The gap is organisational as much as computational: the individual scores already exist in the record, but nothing assembles them, explains them, or connects them to what the relevant guideline recommends, and that assembly work currently falls on the clinician.

Concretely, the decision HepDecide targets is the referral decision in primary and secondary care: whether a patient with abnormal liver biochemistry should be referred for specialist assessment, followed up in primary care, or discharged. Today this decision is made from several indices (FIB-4, APRI, MELD, Child–Pugh) that are computed separately, interpreted against different thresholds, and not linked to the guideline text that justifies the next step. The bottleneck is therefore the assembly and interpretation of fragmented scores within a short consultation, not the absence of any single score. HepDecide does not change the decision itself; it changes the information available for it, presenting one stage-specific estimate, the biomarkers that drove it, and a guideline-traceable narrative in a single view. Whether this alters referral behaviour was not measured here and is the object of the prospective evaluation set out in Section 5.7.

Machine learning (ML) applied to routine laboratory and tabular data has repeatedly outperformed static indices for fibrosis staging and for HCC-risk prediction in chronic liver disease [13,14,15,16]. However, two computational barriers continue to impede the broader clinical adoption of these models. First, most studies deploy a single global model across the entire disease spectrum rather than tailoring separate algorithms to the distinct biochemical characteristics of each stage; stage-specific and multi-task designs have been shown to improve generalisation in liver-disease prediction tasks, including imaging-based ones [17,18]. Second, the black-box nature of high-performing models undermines clinical trust. Post-hoc methods such as SHapley Additive exPlanations (SHAP) quantify each feature’s contribution and are increasingly used to render tabular clinical models transparent [19,20,21]. However, these attributions remain technical in nature and do not independently translate into clinician-interpretable reasoning, leaving a semantic gap between quantitative model output and bedside decision-making [22]. Beyond discriminative performance, a high area under the ROC curve alone does not guarantee clinical usefulness; calibration, decision-analytic benefit, and robustness to class imbalance must also be evaluated [23,24,25].

Large language models (LLMs) offer a route from numerical output to a natural-language clinical narrative, encode substantial medical knowledge [26] and have recently begun to be evaluated for decision support in gastroenterology and hepatology [27]. However, when deployed without grounding mechanisms, LLMs are prone to hallucination, the generation of plausible but factually unsupported content, posing a critical safety risk through the omission or fabrication of clinical guideline recommendations, including drug-dosing information [28,29]. Retrieval-augmented generation (RAG), which grounds model output in an authoritative external corpus, has emerged as a leading approach for improving factual faithfulness in evidence-based clinical text generation [30,31,32]. Beyond safety, translating such systems into practice also requires health-informatics interoperability, that is, standardised data exchange through HL7 FHIR, LOINC, and ICD-10, so that heterogeneous hospital data can be ingested and returned to the EHR without loss of semantic integrity [33,34].

To address these barriers within a single framework, we propose HepDecide, an end-to-end CDSS prototype that spans raw laboratory acquisition to a guideline-grounded, physician-readable report. HepDecide does not introduce a new learning algorithm; its contribution is the system-level integration of components that are usually studied separately, together with a transparent retrospective evaluation of its predictive layer. The framework comprises five elements: (i) stage-specific expert models, one classifier selected independently for each disease stage; (ii) multi-channel data acquisition combining manual entry, optical character recognition, and voice input, designed to reduce clinician data-entry burden; (iii) a hybrid explainability layer that converts SHAP-based quantitative attributions into structured input for narrative generation; (iv) computational grounding, whereby EASL and AASLD guidelines are integrated through a RAG mechanism to reduce unsupported statements; and (v) a standards-oriented output layer, in which the input schema is mapped to LOINC, disease categories to ICD-10, and the three output artefacts are expressed as HL7 FHIR R4 resources. The methodological element is a leakage-controlled retrospective benchmark of the predictive layer on public data, with calibration, decision-curve and explainability analyses, accompanied by a provenance audit of earlier development results which distinguishes recorded estimates from results that can be independently recalculated. We treat interoperability as an explicit design and evaluation dimension rather than as an implementation detail, while making no claim of formal FHIR conformance or live deployment. Section 4.7 sets out the mappings and resource model intended for future conformance and integration testing. The remainder of this paper describes related work, the system architecture and methodology, the retrospective evaluation, the interoperability layer, illustrative case studies, and the study’s limitations.

## 2. Related Work

The informatics of chronic liver disease has evolved rapidly from static statistical scores, through machine learning-assisted prediction, to natural-language interfaces governed by large language models.

### 2.1. Clinical Decision Support Systems and Existing Liver Tools

In routine hepatology, fibrosis and prognostic indices such as FIB-4, APRI, MELD, and the Child–Pugh classification have long served as first-line screening tools [8,9,10,11]. However, rule-based scores that rely on fixed thresholds and a small number of variables are increasingly regarded as insufficient within modern clinical workflows, motivating dynamic, workflow-integrated clinical decision support systems (CDSSs) [35,36]. A recently reported liver CDSS, the LIVERAID-ICU score [37], predicts in-hospital mortality in cirrhotic patients with infections in the intensive care unit. More directly comparable work now covers several subsets of the HepDecide pipeline: HCC treatment and survival prediction [38], hepatology-specific guideline retrieval [39], gastrointestinal RAG-based question answering [40], MASLD fibrosis prediction with SHAP-supported reporting [41], and FHIR-linked guideline RAG outside hepatology [42]. Table 1 makes these overlaps explicit. None of these components is individually new; the remaining distinction is their assembly in one chronic liver workflow and a leakage-controlled retrospective evaluation of the predictive layer. Surveys of decision support also show that technical integration alone does not establish clinical utility [35,36].

### 2.2. Machine Learning for Liver-Disease Staging

Machine learning applied to structured electronic-health-record and tabular data has repeatedly outperformed static indices for fibrosis staging and HCC-risk prediction [13,14,15,16,43,44]; tree-based and gradient-boosting methods are particularly effective for cirrhosis and fibrosis staging, and deep-learning approaches over longitudinal EHR data have been used to anticipate progression to HCC [45]. Most studies, however, deploy a single global model across the entire disease spectrum rather than tailoring algorithms to the distinct biochemical distribution of each stage; stage-specific and multi-task designs have been shown to improve generalisation in related liver-disease prediction tasks [17,18].

### 2.3. Explainability and the Semantic Gap

The black-box nature of high-performing models remains a central barrier to clinical trust [22]. SHAP-based feature-attribution maps are widely used to render tabular clinical models transparent [19,20,21]; yet a semantic gap persists between quantitative explainability and clinical interpretability [22]. HepDecide addresses this gap by passing SHAP attributions directly, as structured input, to a text-generating LLM rather than presenting them as standalone technical plots.

### 2.4. Large Language Models, Retrieval-Augmented Generation, and Standards

LLMs encode substantial clinical knowledge and enable natural-language reporting [26,27], but free-text generation carries the risk of clinical misinformation, and recent work has shown these systems to be vulnerable to adversarially induced hallucination during decision support [46,47]. Retrieval-augmented generation, which grounds outputs in an authoritative external corpus, is the most widely adopted mitigation for evidence-based clinical text generation [30,31,32,48]. Practical deployment additionally relies on parameter-efficient fine-tuning such as LoRA and QLoRA under limited hardware [49,50], on human-aligned text-quality evaluation frameworks [51,52], and on health-informatics interoperability for lossless data exchange with hospital systems [33,34,53].

### 2.5. Positioning and System-Level Contribution

The comparison in Table 1 defines a deliberately bounded contribution. HepDecide is not presented as the first liver CDSS, explainable liver model, hepatology RAG system, or FHIR-linked RAG system. Its contribution is a system-level design that connects patient-level, stage-specific risk estimates to SHAP-derived explanations, guideline-grounded narratives, and standards-oriented outputs in one chronic liver workflow, followed by a retrospective evaluation of the predictive layer in which preprocessing, class balancing and model selection are confined to training data and reported with calibration, decision-curve and explainability analyses. The multi-channel interface further combines manual entry, optical character recognition (OCR) of laboratory documents and on-screen data [54,55], and voice input [56]. The language-generation layer is described as implemented but is not quantitatively evaluated in this paper. This is an integration and evaluation contribution rather than a new algorithmic or clinically validated intervention.

## 3. Materials and Methods

This section describes the study in the order in which the system processes a patient: the design and the data it rests on, the four functional layers of the pipeline, the interoperability layer, and finally the evaluation protocol, which distinguishes the development-phase runs from the leakage-controlled re-analysis that provides the primary results.

### 3.1. Study Design and Overview

**Figure 1:**
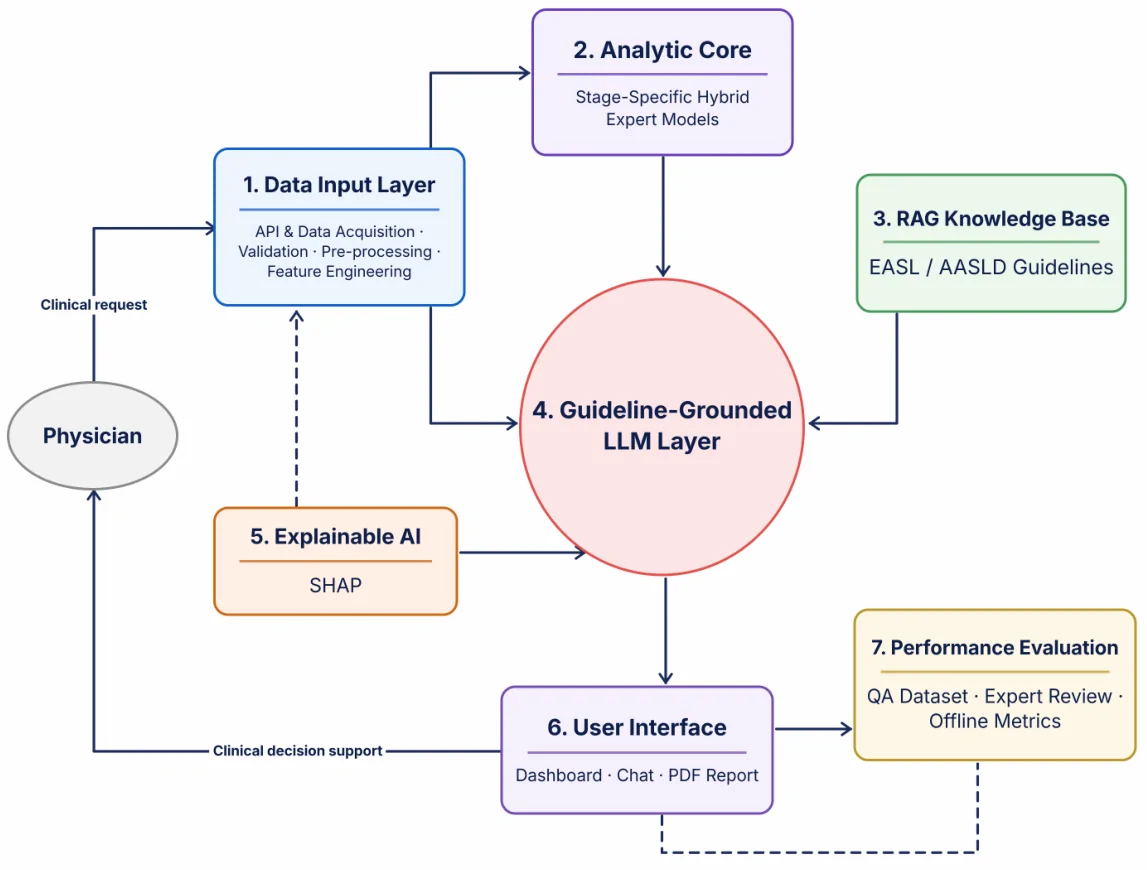
HepDecide system architecture and data flow.

HepDecide is a modular, end-to-end clinical decision support system developed in a retrospective study on publicly available, de-identified secondary datasets. Its purpose is to close the gap between the quantitative risk models used in hepatology and the natural-language reasoning a clinician applies at the bedside. As shown in Figure 1, the platform is organised into seven interoperating modules: the application programming interface (API) and data layer, the analytic core, the RAG knowledge base, the guideline-grounded LLM layer, the explainability (XAI) module, the user-interface layer, and the performance-evaluation module. This study is reported in accordance with the Transparent Reporting of a multivariable prediction model for Individual Prognosis Or Diagnosis + Artificial Intelligence (TRIPOD + AI) reporting guideline for clinical prediction models [57]. Two phases are distinguished throughout. The development phase produced the prototype and a set of archived performance estimates; a subsequent re-analysis, performed during revision on the same public cohorts with a pre-specified leakage-controlled protocol, provides the primary quantitative results (Section 3.8). The present design estimates internal performance within the available source cohorts; performance in independent hospital populations and live clinical workflows requires prospective external evaluation. No patients or members of the public were involved in the design, conduct, or reporting of this study.

### 3.2. Data Sources and Study Population

To develop and internally validate the analytic-core models, we drew on two publicly available, de-identified secondary sources spanning the liver-disease continuum. The first is a biopsy-proven MASLD cohort of 605 patients characterised with the Steatosis–Activity–Fibrosis (SAF) and Kleiner NAS histological scoring systems [58], which supplies both the steatohepatitis label (steatohepatitis versus simple steatosis, as diagnosed by SAF) and the cirrhosis label (histological fibrosis stage 4). The second is the UCI Hepatocellular Carcinoma Survival dataset of 165 patients [59]. Both are open-access, anonymised sources released for academic use; no identifiable patient data were accessed at any stage. The source uses the older NAFLD designation; current MASLD eligibility was not re-adjudicated. The initial cirrhosis experiment used a separate balanced table of 406 records. Its complete source composition, patient uniqueness and overlap with the 605-patient cohort cannot be established from the retained files (File S5).

The MASLD-cohort models used age, sex, AST, ALT, platelets, albumin, body-mass index, INR, total bilirubin, creatinine, direct bilirubin and ALP. The HCC models used age, sex, AST, ALT, albumin, creatinine, INR, platelets, total bilirubin, direct bilirubin, obesity, ALP and AFP. These 12- and 13-variable lists follow the available model interfaces; histology-derived labels were excluded from the predictors. Table 2 and Table 3 describe the data before imputation or resampling, and Table 4 is an interface terminology schema, not a complete model feature list. Source values were retained without undocumented unit conversion. In particular, the HCC platelet column has incompatible apparent scales and inconsistent unit documentation; its use as recorded limits interpretation and deployment.

The cirrhosis subgroup in Table 2 is nested within the MASLD cohort. Values for the MASLD cohort and for that subgroup were computed directly from the source dataset [58]. The international normalised ratio and creatinine were recorded for 304 and 305 patients respectively (20 of the 24 F4 patients), so the corresponding statistics are based on those patients, and a dash marks a variable that was not tabulated for the cohort concerned. HCC statistics were recalculated from available observations in the 165-row source. Its platelet values range from 1.71 to 459,000 despite a single G/L label, so a common-unit mean is not reported. Albumin is shown on the recorded numerical scale because its repository unit label is inconsistent. Variable-specific counts and ranges are supplied in File S6.

Cohort definition and representativeness. No inclusion or exclusion criteria were applied beyond those inherent to the source repositories. The MASLD cohort [58] is a publicly released, biopsy-scored dataset of 605 patients with SAF and Kleiner NAS histology; its originating institution, recruitment period and ethical approval are not documented by the repository, which limits what can be stated about the underlying population. Sex was recorded for all patients (284 female, 321 male); 225 (37.2%) had diabetes and 392 (64.8%) met metabolic-syndrome criteria as coded in the source. Before class balancing, the steatohepatitis label was positive in 537 of 605 patients (88.8%), any fibrosis (F1–F4) was present in 409 (67.6%), and cirrhosis (F4) in 24 (4.0%) (Table 3). The HCC cohort [59] was collected at a Portuguese university hospital and comprises 165 patients with an established diagnosis of HCC (32 female, 133 male; age 64.7 ± 13.3 years); cirrhosis was recorded in 149 (90.3%). Its label is one-year survival (63 died, 102 survived; 38.2% event rate). Missing data represent 10.2% of the HCC cohort and only eight patients (4.8%) are complete on all 49 source fields. The HCC-stage model therefore learns to separate poor-prognosis from better-prognosis HCC on the shared biochemical schema, and does not estimate the probability of having HCC. Survival is the positive class in the re-analysis. Sample size was determined by the available records, without a formal calculation. Recruitment dates, assessor details and blinding, measurement timing and treatment adjustment were not sufficiently documented in the available analysis records.

Neither cohort represents the intended point-of-use population. Both are specialist, biopsy- or oncology-referred series: steatohepatitis is present in 88.8% of the MASLD cohort, cirrhosis in 4.0%, and HCC in 100% of the HCC cohort, whereas an unselected primary-care population has far lower prevalence of every stage and contains HCC-negative patients that the HCC cohort does not. The two cohorts also overlap clinically, since nine in ten HCC patients are cirrhotic, while having incompletely documented recruitment settings and periods; this is one reason between-stage comparisons should be read cautiously (Section 5.6). The minority classes are small in absolute terms (68 simple-steatosis and 24 F4 patients), which is why class balancing was required and why the event counts in the held-out partitions are modest. Discrimination estimates reported here therefore describe separation within referred populations; positive predictive value in a low-prevalence setting would be substantially lower, and no claim is made about screening performance.

Disease categories were aligned to ICD-10 as follows: MASLD (K76.0/K75.8), cirrhosis (K74.6) and HCC (C22.0).

### 3.3. API and Data Layer: Acquisition and Preprocessing

The API and data layer ingests heterogeneous clinical inputs and transforms them into a harmonised, model-ready representation for the analytic core. To reduce clinician data-entry burden, it accepts three input channels: (i) manual entry; (ii) optical character recognition of laboratory reports using Tesseract [60] and Google Gemini; and (iii) a voice-command assistant based on the Web Speech API. A Flask-based interface standardises these inputs and persists them to a PostgreSQL database (accessed through psycopg2-binary 2.9.7; the server version was not recorded in the archived configuration).

Harmonisation proceeded in three stages. First, semantic feature alignment: variables represented under different names across sources (e.g., plt, platelets) were mapped to standard feature names through a custom ontological dictionary developed for this study. In the re-analysis, source values were retained without undocumented unit conversion or z-score exclusion. Missing values were imputed with k-nearest-neighbours imputation (k = 5), and scaling was applied where required by the classifier. KNN imputation was chosen because it preserves the multivariate structure among correlated liver biomarkers (for example AST, ALT and GGT) rather than collapsing each field to a marginal constant, and because it handles the block-wise missingness of INR and creatinine in the MASLD cohort without discarding half of that cohort. Its sensitivity was assessed against median imputation in the re-analysis (Section 4.2). Because KNN distances are computed on the pre-scaled variables, the influence of variable units on neighbour selection is a recognised limitation.

The point at which preprocessing was fitted differs between the two phases and is stated explicitly because it affects the risk of information leakage. In the MASLD development run, the scaler used all 605 records. HCC class balancing preceded splitting, but its scaler matches the reconstructed training partition. In the initial cirrhosis experiment, BMI was imputed by a single-column KNN procedure and INR and creatinine by their medians before splitting; XGBoost used unscaled predictors, whereas scaling for other classifiers was fitted after splitting. These procedures can introduce optimism, although its magnitude cannot be determined from the retained records (File S5). In the re-analysis, the data were first partitioned patient-wise into an 80% training set and a 20% locked test set (stratified by endpoint), and imputation, standardisation, resampling and calibration were fitted exclusively on the training data, inside each of five cross-validation folds during model selection and on the full training set for the final model. The locked test set was scored once, after all model-selection decisions had been fixed.

### 3.4. Analytic Core: Stage-Specific Hybrid Expert Models

**Figure 2:**
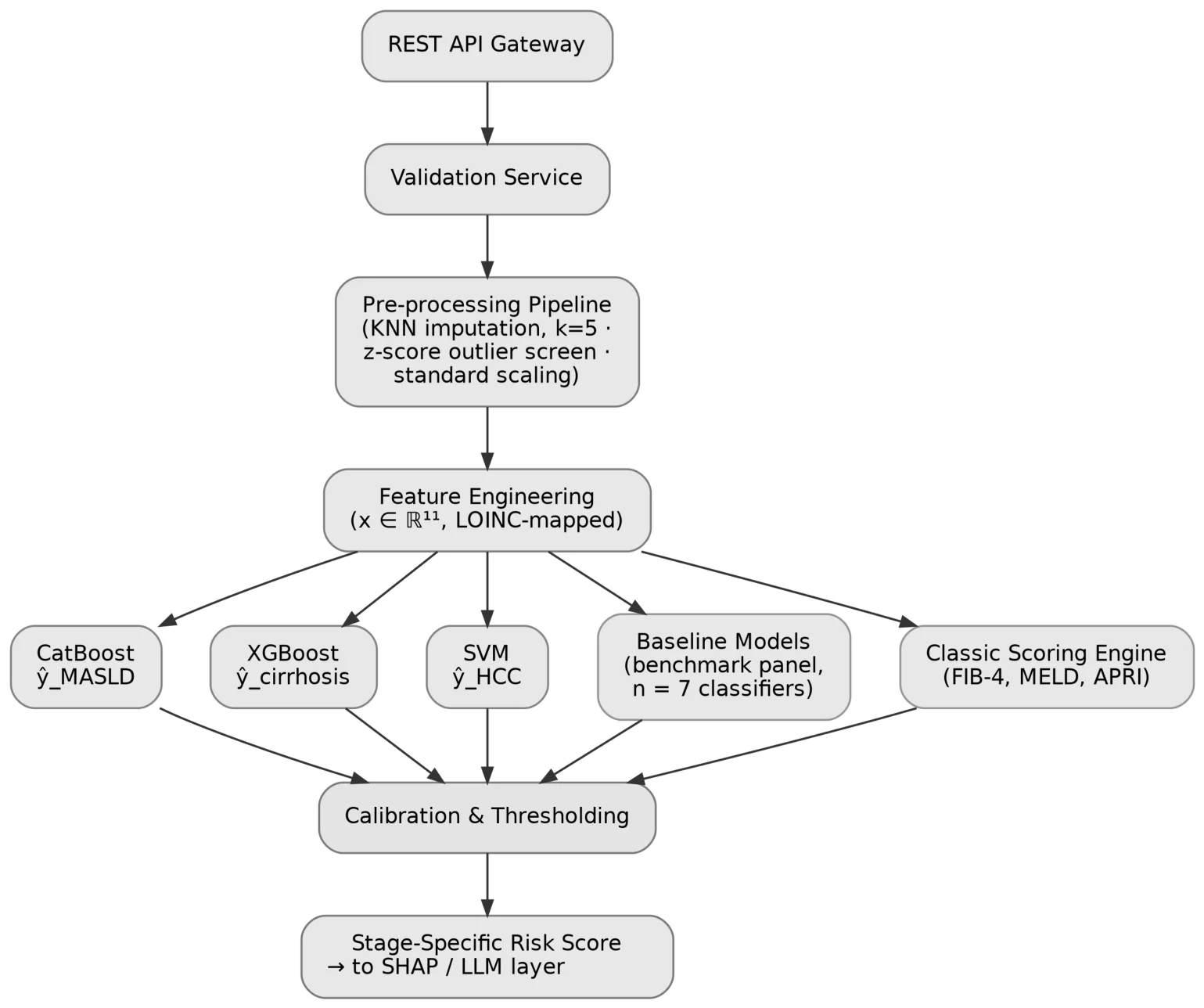
Data-processing pipeline and the three stage-specific hybrid expert models as configured in the deployed prototype. The classifiers selected in the leakage-controlled re-analysis are given in Section 3.4.

The analytic core performs the quantitative risk estimation central to HepDecide, producing a stage-specific risk for each patient. Rather than a single global model, an independently optimised classifier is constructed for each disease stage. In the development phase, CatBoost [61] was selected for the MASLD stage, XGBoost [62] for cirrhosis and a support vector machine (SVM) [63] for HCC, and these are the algorithms wired into the deployed prototype (Figure 2). In the re-analysis, five classifiers (logistic regression, random forest [64], CatBoost, XGBoost and an RBF-kernel SVM) were each combined with five class-imbalance strategies (none, class weighting, random over-sampling, random under-sampling and SMOTE [65]), giving 25 configurations per endpoint; every configuration was scored by mean ROC-AUC under five-fold stratified cross-validation on the training set, and the best configuration was locked before the test set was scored. The selected configurations were class-weighted CatBoost for steatohepatitis, random over-sampled random forest for cirrhosis and random over-sampled random forest for one-year HCC survival. Random resampling was retained as a candidate strategy, alongside class weighting and SMOTE, because the smallest minority classes (24 F4 and 68 simple-steatosis patients) make synthetic interpolation unreliable in the tails of the biomarker distributions and because class weighting is not implemented uniformly across the benchmarked algorithms; the comparison of all five strategies is reported in Section 4.2. Algorithm settings were fixed in this re-analysis; the initial hyperparameter search was not repeated. For the XGBoost class-weight candidate, the weight ratio used the enclosing training set rather than each inner fold, without using test labels. None of the three selected models used that candidate.

### 3.5. Explainability (XAI) Module

The explainability module makes the analytic core’s predictions transparent and clinically interpretable, so that risk estimates are consistently accompanied by patient-specific explanatory context rather than presented as an opaque number. Model transparency was provided by SHAP [19]. In the additional analyses, TreeSHAP [66] was used for tree models, LinearExplainer for logistic regression and permutation SHAP for the SVM; SHAP decomposes each prediction into additive feature attributions ($\hat{y}=\varphi_{0}+\sum_{i}\varphi_{i}$), where $\varphi_{i}$ is the Shapley value representing the contribution of feature *i* to the prediction. For each patient, the module produced the top five dominant biomarker contributors, ranked by absolute SHAP magnitude and each flagged relative to its clinical reference range (above, within, or below normal). Five contributors were retained because this is small enough to fit a single dashboard panel and a one-sentence narrative, and because in the re-analysis the five highest-ranked features accounted for 58–73% of the total mean absolute SHAP attribution for the three models (Section 4.4); the complete attribution profiles are nevertheless reported for all models. This structured output, rather than a raw probability, was passed to the guideline-grounded LLM layer. These attributions explain the uncalibrated base estimators. Tree models used their path-dependent reference distribution; logistic regression and SVM used 32 training records as background, with ten permutations for the SVM. Output scales were probability for random forests and SVMs, log odds for logistic regression and raw margin for boosted trees. Arrays and method details for all 15 endpoint–algorithm combinations are provided in File S6.

### 3.6. Guideline-Grounded LLM Layer

**Figure 3:**
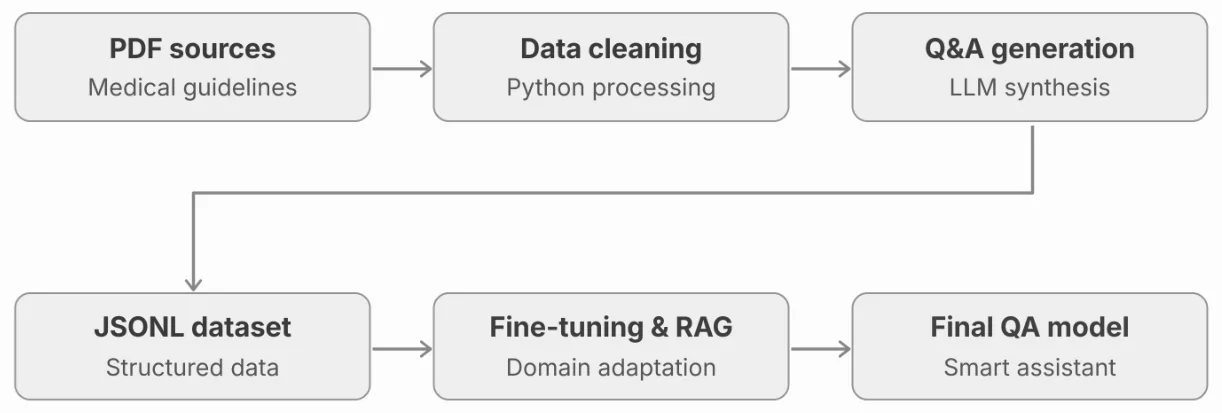
LLM training-data preparation and model-development workflow, from guideline sources to the guideline-grounded clinical assistant, as proposed during development. The available training run is specified in Section 3.6 and File S4; the diagram does not establish clinical quality control or a held-out evaluation.

The guideline-grounded LLM layer is the component that most distinguishes the pipeline from a stand-alone classifier: it converts the quantitative outputs into a natural-language, evidence-oriented clinical narrative while constraining generation to retrieved guideline content. During development, the interpretation model was built on the Llama-3.1-8B-Instruct foundation model [67] and domain-adapted with Quantized Low-Rank Adaptation (QLoRA) [49,50] over a corpus of curated medical question–answer pairs generated from guideline documents (Figure 3). The recovered corpus contains 4104 system–user–assistant records. The recorded configuration used a maximum sequence length of 8192, LoRA rank 64, alpha 128, dropout 0, learning rate 2 × 10^4^, batch size 4, gradient accumulation 4 and seed 3407. Training ran for five epochs and 1285 optimisation steps. All records were used for training; no validation split or early stopping was defined. Messages were formatted with the tokenizer chat template, with a system instruction to answer using the supplied medical context. Explicit source filenames occur in 132 records and identify 33 files; the other 3972 records lack that filename marker, although they can contain context text. Annotation authorship, complete source mapping and clinical quality-control records were unavailable. The retained 128-point training log and corpus audit are reported in File S4.

**Figure 4:**
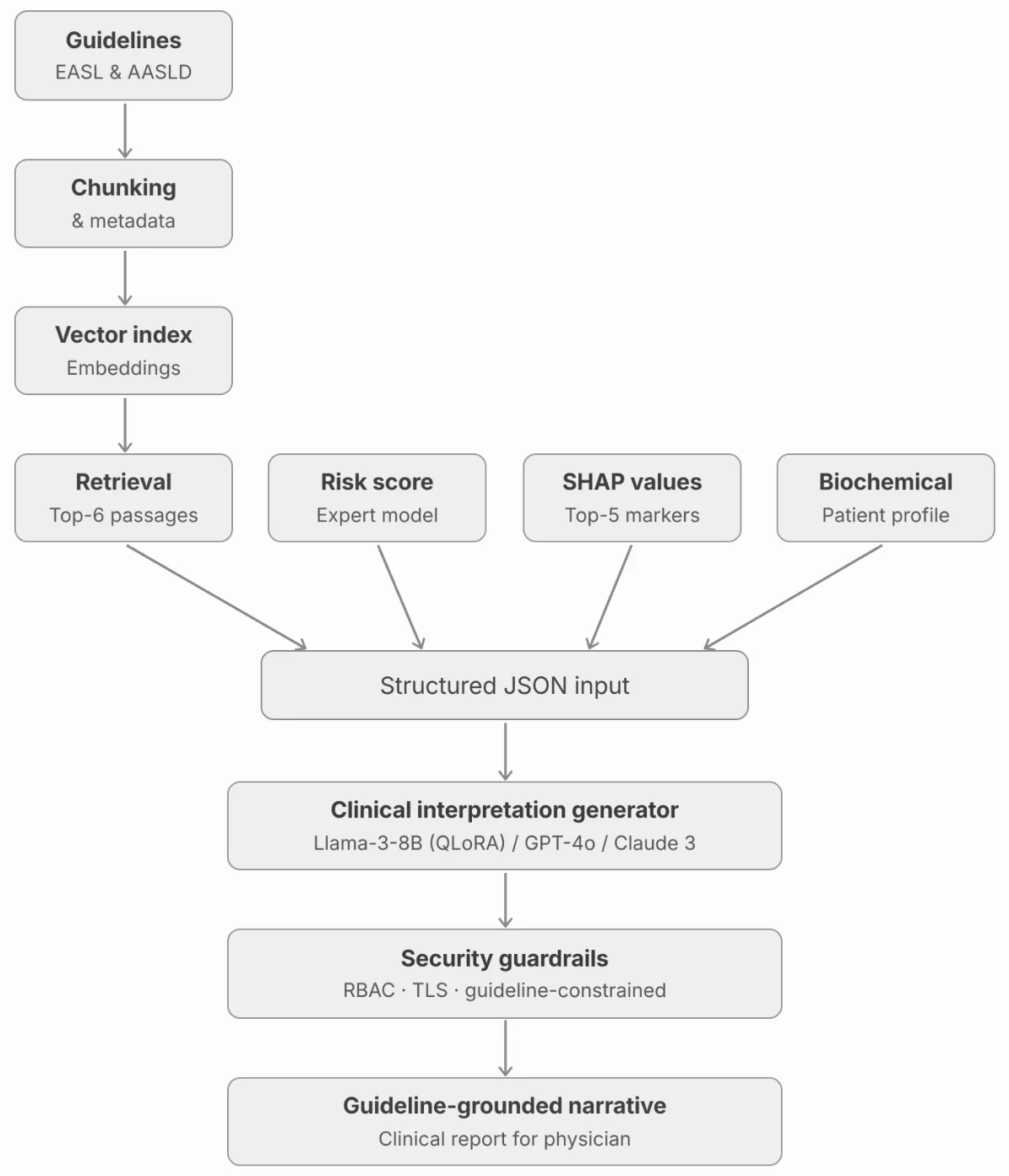
Retrieval-augmented generation and guideline-grounded interpretation pipeline (schematic of the design). The retrieval parameters of the released code are given in Section 3.6. The top-six retrieval shown is the earlier design; the available implementation uses top-two retrieval (Section 3.6).

To ground generation in authoritative evidence, a retrieval-augmented generation mechanism [30] retrieves guideline passages for each query and combines them with the expert-model risk scores, the SHAP attributions, and the patient’s biochemical profile in a structured JavaScript Object Notation (JSON) prompt (Figure 4). The retrieval configuration in the released code is as follows: the repository contains 36 PDFs and three text files; extracted text is chunked into 800-character passages with a 100-character overlap, embedded with the BAAI/bge-m3 model [68], and ranked by cosine similarity, with the two highest-ranked passages passed to the generator; no re-ranking stage is implemented, and retrieval latency was not measured separately. The design target is a corpus restricted to the EASL and AASLD clinical practice guidelines [5,6,69,70]; the repository also contains supporting reference material, and restricting the corpus is listed as future work. Faithfulness of generated text to the retrieved context can be assessed with the RAGAS framework [71], factual precision with FactScore [72] and semantic similarity with BERTScore [73]; these metrics were computed during development but the query-level outputs were not retained, so no text-quality result is reported here. In the prototype interface, guideline-grounded generation can additionally be performed through instruction-tuned commercial LLMs, GPT-4o and Claude 3, accessed via API and constrained by the same retrieved evidence [74,75]; these back-ends are interchangeable implementation options bound by the same retrieved context. The training notebook reports 3546 PDF-derived chunks after excluding passages of 100 characters or fewer. The index itself was not supplied, so exact membership cannot be confirmed. The earlier FAISS/top-six configuration cannot be linked to the reported language evaluation (File S4).

### 3.7. Health-Informatics Standards, User Interface, and Prototype Implementation

**Figure 5:**
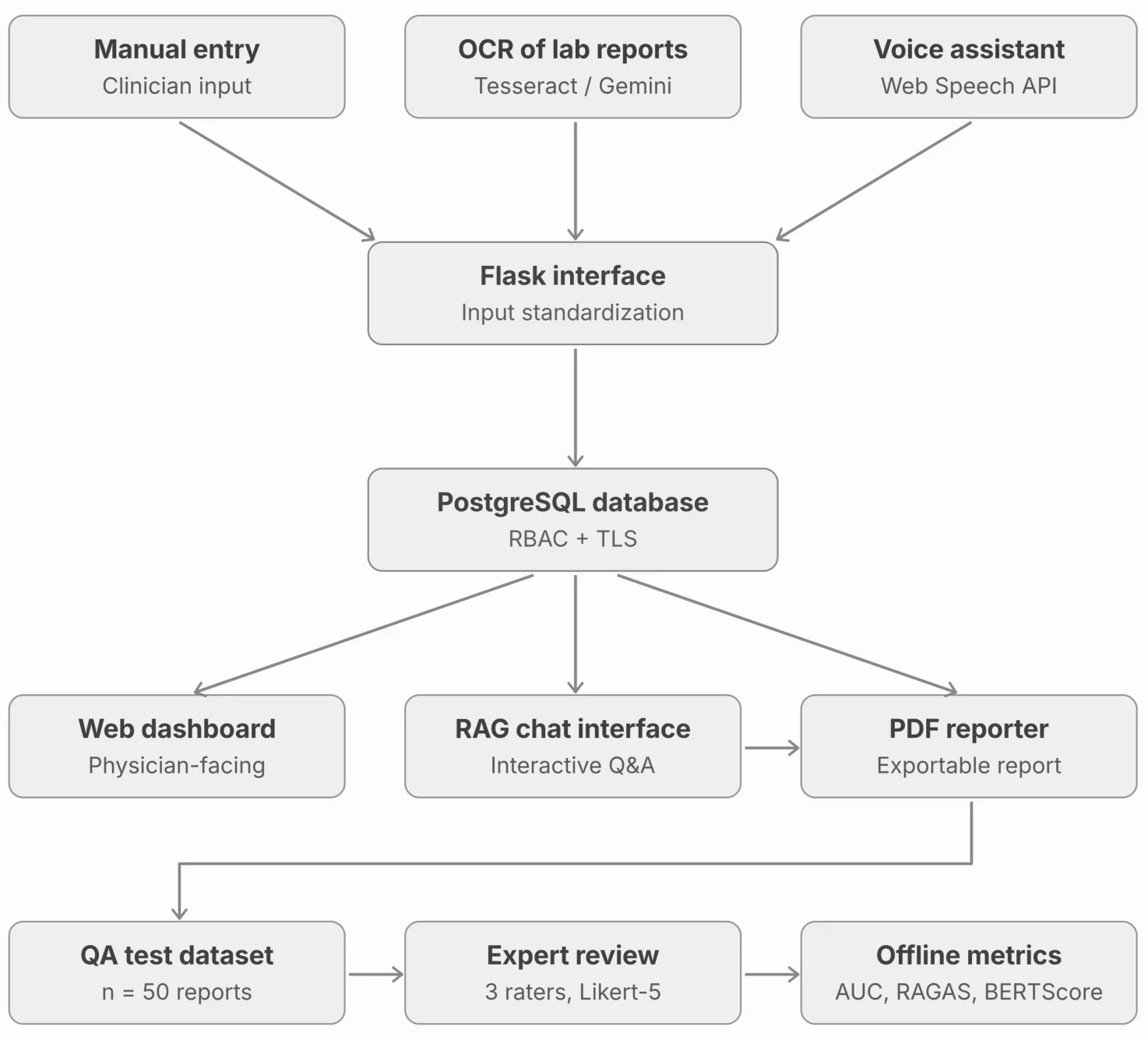
Multi-channel data acquisition, physician-facing interface and the closed performance-evaluation loop. The expert-review and offline text-metric elements of the loop represent the intended evaluation workflow; retained results do not verify their completion (Section 3.8).

The user-interface layer delivers the system’s outputs to clinicians within their workflow. It consolidates the stage-specific risks, the SHAP-based explanations, and the guideline-grounded narrative into a single physician-facing dashboard, complemented by an interactive RAG chat interface and an exportable PDF clinical report. The multi-channel input pathways, the physician-facing interface, and the closed evaluation loop are shown in Figure 5.

For interoperability, the eleven fields of the input schema were mapped to LOINC codes (Table 4) and disease categories aligned to the International Classification of Diseases, 10th revision (ICD-10), preserving semantic identity when differently named fields are represented in the prototype [33,34,53]. The prototype data model follows the Health Level Seven Fast Healthcare Interoperability Resources (HL7 FHIR) Release 4 standard: laboratory inputs are formatted as Observation resources, model-generated risk scores as RiskAssessment resources, and the final RAG-generated clinical narrative as a DiagnosticReport resource intended for future transmission to hospital information systems. FHIR-formatted packets are processed asynchronously in JSON format via RESTful API endpoints.

To safeguard patient data, the architecture specifies proposed technical controls intended to support both the Turkish Personal Data Protection Law (KVKK) and the United States Health Insurance Portability and Accountability Act (HIPAA) Security Rule. The proposed design encrypts data at rest using AES-256 and data in transit are protected by TLS 1.3. The design provisions API access through role-based authentication with OAuth 2.0 and JSON Web Token authorisation, and specifies that every prediction request is anonymised and written to an immutable audit log. Formal FHIR conformance testing and multi-site clinical deployment were outside the scope of the present study and are left as future work. Implementation and effectiveness of these controls were not verified in the retrospective analysis.

### 3.8. Performance Evaluation, Statistical Analysis, and Software

The evaluation reported in this paper concerns the predictive layer and is organised in two parts. Development-phase estimates. During development, each stage-specific model was benchmarked on a held-out partition and an ROC-AUC was recorded; the saved model, imputer and scaler files, derivative data tables and the application source code were archived in a public repository. Before revision, these artefacts were audited to establish, for each archived estimate, whether the model, data partition and endpoint could be reconstructed; the outcome of that audit is reported in Section 4.6 and determines which archived values are retained. Re-analysis (primary analysis). Because complete patient-level predictions and SHAP arrays from the development runs were not retained, a separate retrospective re-analysis of the same two public cohorts was performed during revision under a pre-specified, leakage-controlled protocol (random seed 20260920; not preregistered). For each endpoint the cohort was split patient-wise into 80% training and 20% locked test sets, stratified by outcome. Within the training set, 25 classifier–imbalance configurations (Section 3.4) were compared by five-fold stratified cross-validation, with KNN imputation, standardisation and resampling fitted inside each fold; median imputation was run as a sensitivity analysis. The selected configuration was refitted on the full training set, its probabilities were calibrated by sigmoid (Platt) scaling using five-fold cross-validation on training data only, and the locked test set was scored once. Reported metrics are ROC-AUC, average precision, Brier score, and the confusion matrix, sensitivity and specificity at a fixed 0.5 probability threshold, with 95% percentile bootstrap intervals for ROC-AUC, average precision and Brier score (2000 test-set resamples, omitting undefined single-class draws; the bootstrap was preferred to the DeLong method [76] because of the small numbers of events), and exact binomial intervals for sensitivity and specificity in File S6. Calibration curves are shown before and after calibration. Decision-curve analysis [23] was computed over threshold probabilities from 0.05 to 0.95 against the treat-all and treat-none strategies; because no intervention or clinically acceptable threshold range was specified before analysis, these curves are reported as exploratory. Explainability was computed for the training-set base estimators using the methods in Section 3.5, with the full attribution profile and the cumulative share of the five highest-ranked features reported for every model. On the cirrhosis test partition, FIB-4 and APRI were computed from the source variables at their conventional thresholds (3.25 and 1.0) for a like-for-like comparison. Language layer and expert review. A blinded rating exercise of generated reports and automated text-quality metrics were carried out during development; the rater records, rated cases and query-level metric outputs were not retained, so no result from that exercise is reported (Section 5.6 and Section 5.7). Calibration used an ensemble of estimator–calibrator pairs fitted within training folds. Constant-training prevalence supplied the Brier reference. APRI used an AST upper limit of 40 U/L and platelets in 10^9^/L; the index calculations were independent of the archived application.

All analyses were implemented in Python (version 3.11). The re-analysis environment (File S2) used scikit-learn 1.7.0, imbalanced-learn 0.14.2, CatBoost 1.2.8, XGBoost 3.0.2, SHAP 0.51.0, pandas 2.3.1, NumPy 2.3.1, SciPy 1.16.0 and Matplotlib 3.10.3. The development-phase application used scikit-learn 1.3.0, CatBoost 1.2.8, pandas 2.0.3 and NumPy 1.24.3, with Flask 2.3.3 and a PostgreSQL backend accessed through psycopg2 2.9.7, and Tesseract for OCR. The LLM fine-tuning used the Hugging Face Transformers and PEFT (QLoRA) libraries. The re-analysis code, its unit tests, pinned requirements, the SHA-256 hashes of the source files, the patient-level test predictions and all figure-generating outputs are provided as Supplementary Material to reproduce the re-analysis results; the limits of reconstructing development-phase values are stated in Section 4.6. This study was not preregistered, and no analysis plan was registered in advance. Generative artificial-intelligence assistants were used during the preparation of this manuscript, for drafting and language editing of the text and for support in configuring parts of the data-processing and generation pipeline code; the tools are identified in the Acknowledgments. Numerical results were calculated by the accompanying scripts. No generated patients were added to the source test cohorts. Archived estimates are identified separately, and the author is responsible for the text, analysis and interpretation.

### 3.9. Additional Internal Analyses (Group B)

Group B provides the additional diagnostic analyses requested during review. Its partitions, model selection and cohort definitions differ from the initial experiments; it is not a reconstruction of their reported AUCs. The three selected-model predictions were frozen before the extension. To provide plots for every algorithm, the imbalance strategy for each of the five algorithms was selected by training cross-validated AUC, then average precision; remaining ties followed a recorded strategy order. All 15 endpoint–algorithm combinations were evaluated on their existing test partitions. A separate cirrhosis sensitivity analysis used five stratified outer folds, with three inner folds to select among 25 configurations and three training-only folds for sigmoid calibration. KNN was fixed. Every source record received one outer-fold prediction, including all 24 F4 cases. The outer models have overlapping training sets; pooled results are descriptive and no confidence interval treating the folds as independent studies was calculated. Protocols and outputs are in File S6.

## 4. Results

Section 4.1, Section 4.2, Section 4.3, Section 4.4 and Section 4.5 report the leakage-controlled re-analysis, which is the primary quantitative evaluation of this paper. Section 4.6 reports the provenance audit of the archived development-phase estimates and states which are retained. Section 4.7 reports the interoperability design, and Section 4.8 closes with two illustrative cases showing the prototype end to end.

### 4.1. Discrimination, Calibration and Classification Performance (Re-Analysis)

**Figure 6:**
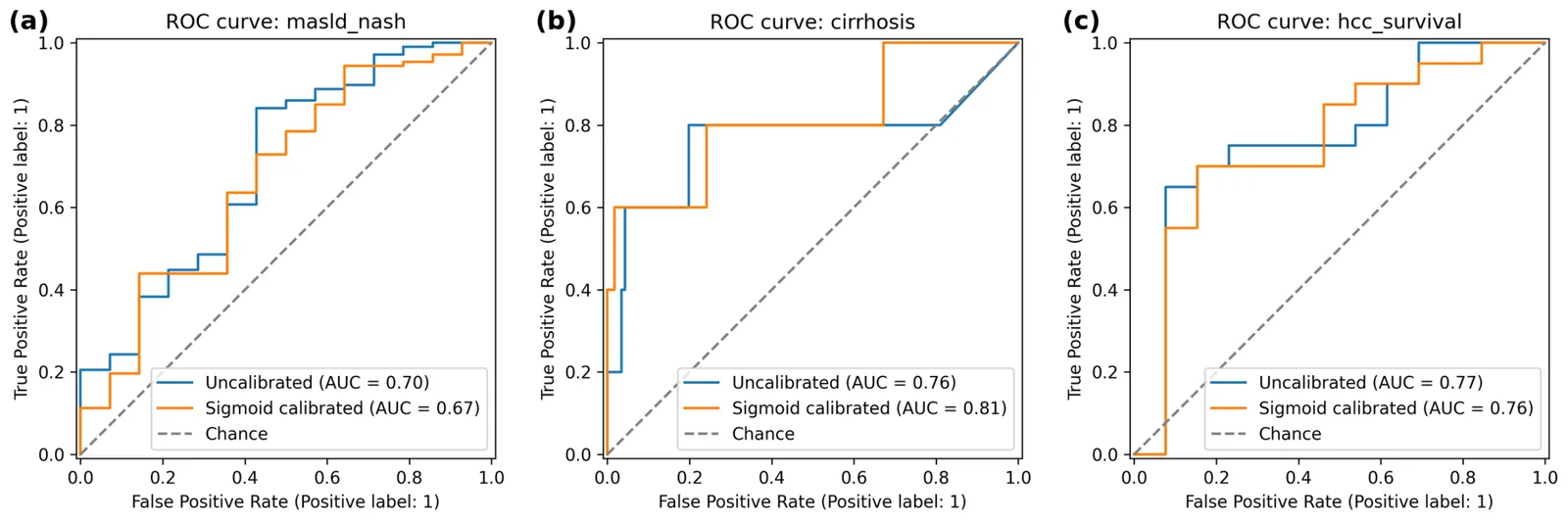
Receiver operating characteristic curves of the selected configuration on the locked test partition, re-analysis: (**a**) steatohepatitis versus simple steatosis, (**b**) histological cirrhosis, (**c**) one-year survival in diagnosed HCC.

**Figure 7:**
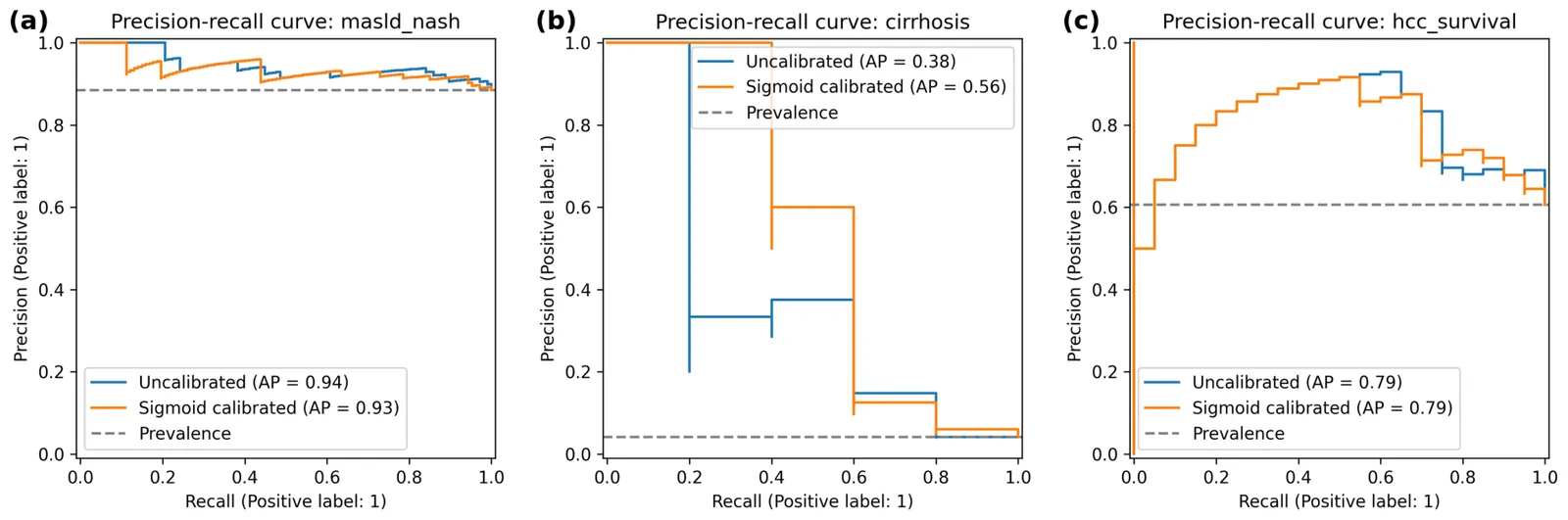
(**a**–**c**) Precision–recall curves on the locked test partitions, re-analysis; panels as in Figure 6. The dashed line marks the test-set prevalence.

**Figure 8:**
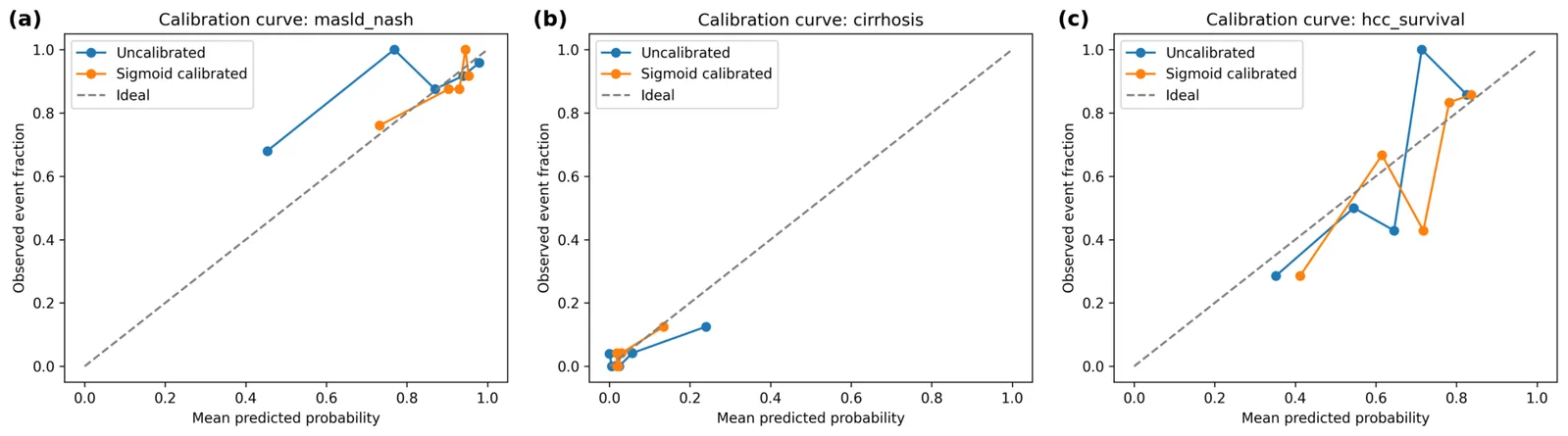
(**a**–**c**) Calibration curves before and after sigmoid calibration on the locked test partitions, re-analysis; panels as in Figure 6. Calibration was fitted on training data only.

Table 5 reports the performance of the three selected configurations on their locked test partitions; the corresponding receiver operating characteristic and precision–recall curves are shown in Figure 6 and Figure 7, and the calibration curves in Figure 8; the confusion matrices at the fixed 0.5 threshold are shown in Section 4.3. Discrimination was moderate for all three endpoints and every confidence interval was wide. For steatohepatitis versus simple steatosis (121 test patients, 107 events) the calibrated class-weighted CatBoost model reached an ROC-AUC of 0.67 (95% CI 0.50–0.84) and a Brier score of 0.096; because 88% of the test patients were positive, the fixed 0.5 threshold classified every patient as positive (sensitivity 1.00, specificity 0.00), so the average precision of 0.93 chiefly reflects prevalence and the model adds little over the base rate at that threshold. For histological cirrhosis (121 test patients, five events) the random over-sampled random forest reached an ROC-AUC of 0.81 (0.52–1.00) and a Brier score of 0.028; at the 0.5 threshold it identified one of the five F4 patients with no false positives (sensitivity 0.20, specificity 1.00). For one-year survival among patients with HCC (33 test patients, 20 survivors) the random over-sampled random forest reached an ROC-AUC of 0.76 (0.56–0.92) and a Brier score of 0.199, with sensitivity 0.95 and specificity 0.31 at the 0.5 threshold. Cross-validated ROC-AUC on the training sets was higher than the locked test values for the two MASLD-cohort endpoints (0.76 ± 0.07 and 0.91 ± 0.06 for steatohepatitis and cirrhosis), which is the expected direction of optimism for small event counts, and similar for HCC survival (0.80 ± 0.07).

Calibration (Figure 8) appeared close to the diagonal for the cirrhosis model in the low-probability range in which almost all test patients fall, and the sigmoid step reduced over-confidence of the steatohepatitis model in its lowest-probability bin; for HCC survival the calibrated curve remained irregular, reflecting the 33-patient test set. Sigmoid calibration changed the Brier score by at most 0.016 and changed ROC-AUC in either direction by up to 0.05, because this comparison is between a single base fit and a cross-validated calibrated ensemble, rather than a single monotone transformation of the same predictions. Constant-training-prevalence Brier scores were 0.102, 0.040 and 0.239, giving Brier skill scores of 0.062, 0.282 and 0.167. The small test sets do not establish calibration in another population.

In the additional five-fold outer evaluation, all 605 records received a held-out prediction, including all 24 F4 cases. Pooled ROC-AUC was 0.819, average precision 0.256 and Brier score 0.034; fold AUCs ranged from 0.779 to 0.953. At threshold 0.5, TN/FP/FN/TP were 578/3/22/2. This is an internal sensitivity analysis of the selection procedure, not a reproduction of the initial 0.98 estimate (File S6).

### 4.2. Imputation and Class-Imbalance Sensitivity

Table 6 reports the sensitivity of the locked test results to the imputation method and, for the selected classifier of each endpoint, the cross-validated discrimination obtained with each of the five class-imbalance strategies. Replacing KNN by median imputation changed the test ROC-AUC by +0.02, –0.02 and +0.03 for steatohepatitis, cirrhosis and HCC survival respectively, and the Brier score by no more than 0.010; the choice of imputer therefore did not alter any conclusion. Among the imbalance strategies, class weighting, random over-sampling and no correction produced cross-validated ROC-AUC values within 0.02 of one another for all three endpoints, SMOTE was never superior, and random under-sampling was consistently the weakest, by 0.04–0.06, because it discards most of the majority class in cohorts that are already small. The strategy selected by the pre-specified rule (highest mean cross-validated ROC-AUC) was class weighting for steatohepatitis and random over-sampling for the other two endpoints; the best XGBoost or SVM configuration was within 0.03 of the selected configuration for every endpoint, so the choice among the tree-based and kernel methods was not decisive on these data.

### 4.3. Decision-Curve Analysis (Exploratory)

**Figure 9:**
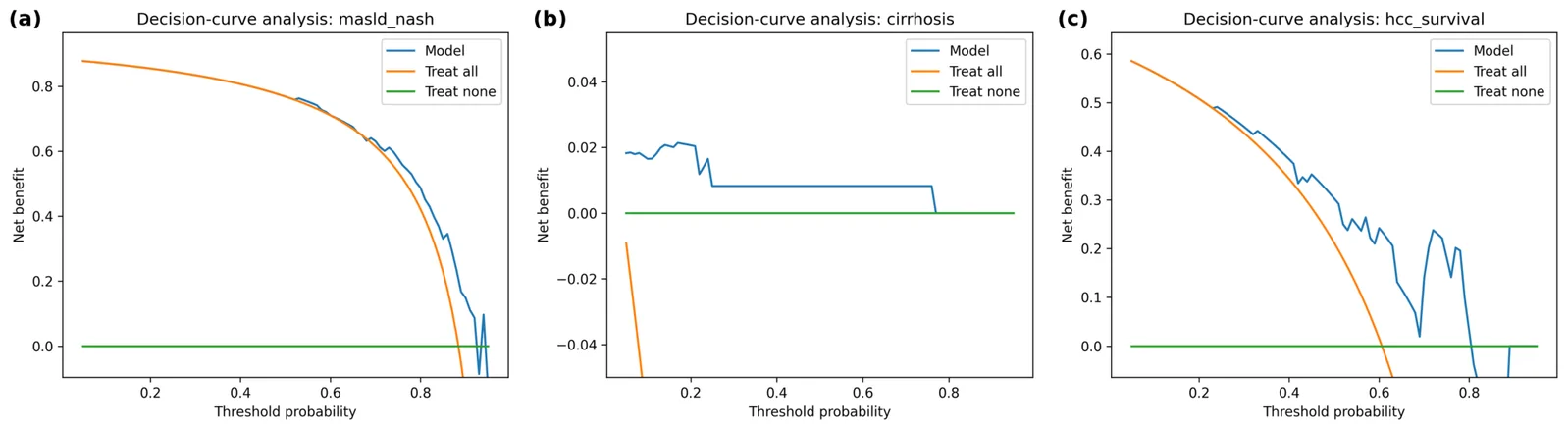
(**a**–**c**) Exploratory decision curves of the selected configuration on the locked test partitions, re-analysis; panels as in Figure 6. Net benefit is shown against the treat-all and treat-none strategies.

**Figure 10:**
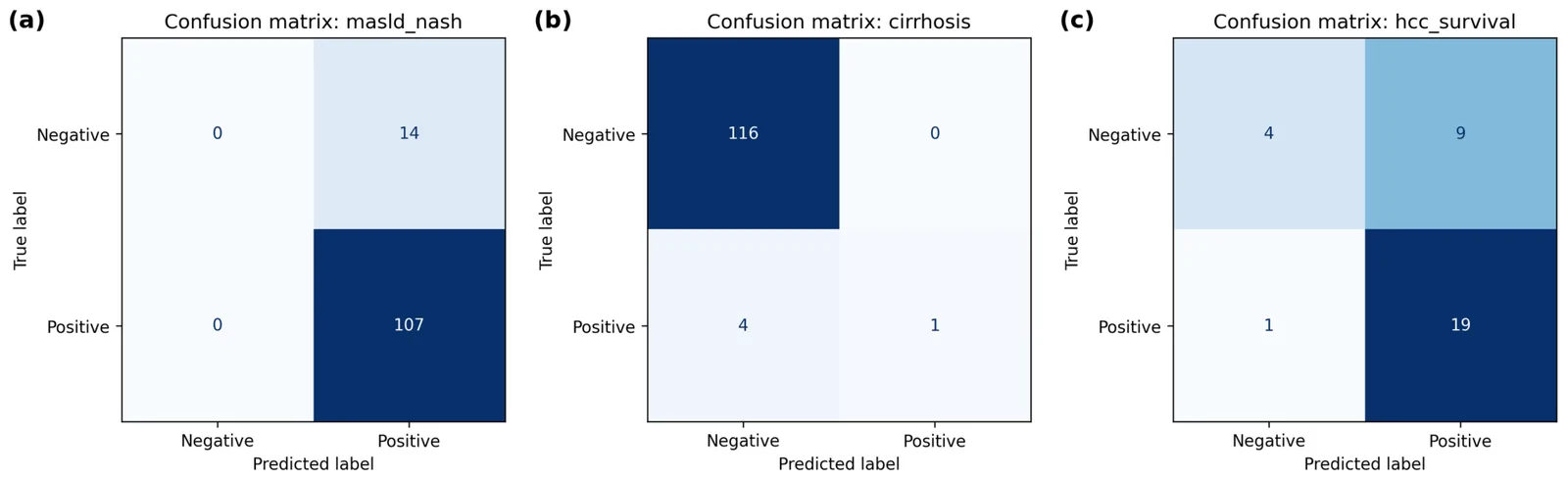
(**a**–**c**) Confusion matrices of the selected configuration at the fixed 0.5 probability threshold on the locked test partitions, re-analysis; panels as in Figure 6.

Figure 9 shows the net benefit of each selected model against the treat-all and treat-none strategies over threshold probabilities from 0.05 to 0.95. The cirrhosis model exceeded both default strategies over thresholds of 0.05–0.76, the HCC-survival model over 0.24–0.80, and the steatohepatitis model only over 0.69–0.92. These curves are exploratory: no intervention, harm–benefit ratio or clinically acceptable threshold range was pre-specified, the cirrhosis curve rests on five events, and for the HCC endpoint the positive class denotes survival, so the curve does not correspond to a treatment decision. They are reported to satisfy the methodological expectation that a clinical prediction model be assessed for decision-analytic benefit [23,24,25], not as evidence of clinical usefulness. The confusion matrices of the same three models at the fixed 0.5 probability threshold are shown in Figure 10.

### 4.4. Explainability: SHAP Attribution for All Models

**Figure 11:**
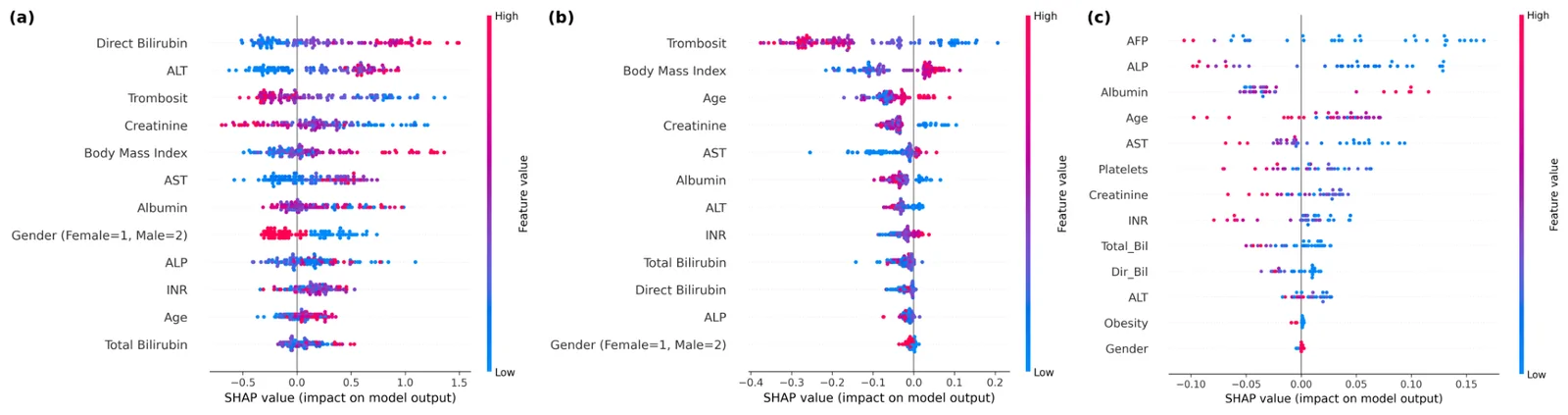
SHAP summary plots of the selected model for each endpoint, re-analysis: (**a**) steatohepatitis versus simple steatosis (CatBoost), (**b**) histological cirrhosis (random forest), (**c**) one-year survival in diagnosed HCC (random forest). All input features are shown, ranked by mean absolute SHAP value.

Figure 11 shows the SHAP summary plots for the three selected models, and the five highest-ranked features with their cumulative share of total mean absolute attribution are as follows. For steatohepatitis they are direct bilirubin, ALT, platelet count, creatinine and body-mass index (57.9% of total attribution). For cirrhosis they are platelet count, body-mass index, age, creatinine and AST (72.6%). For one-year HCC survival they are alpha-fetoprotein, alkaline phosphatase, albumin, age and AST (69.0%); sex and obesity status together contributed less than 1%. The cirrhosis ranking, dominated by platelet count, and the HCC ranking, led by alpha-fetoprotein and albumin, reproduce established clinical expectation and thereby support the plausibility of the explanations surfaced to the clinician; the steatohepatitis ranking is more diffuse, consistent with the weaker discrimination of that model. Two points of scope apply. The attributions explain the base model refitted on the training set, not the cross-validated calibrated ensemble. And the feature set of the HCC model additionally includes direct bilirubin, sex and obesity status and does not include gamma-glutamyl transferase, because those are the fields available in that cohort (Section 3.2). File S6 supplies corresponding plots and numerical attributions for all five algorithms across the three endpoints.

### 4.5. Comparison with FIB-4 and APRI on the Cirrhosis Test Partition

On the same 121-patient locked cirrhosis test partition, FIB-4 and APRI were computed from the source variables (Table 7). FIB-4 reached an ROC-AUC of 0.92 (95% CI 0.82–0.99) and APRI 0.83 (0.69–0.97), against 0.81 (0.52–1.00) for the re-analysis random forest model; the confidence intervals overlap widely, and with five F4 events no ordering among the three can be asserted. At the conventional thresholds, FIB-4 > 3.25 identified two of the five F4 patients with two false positives and APRI > 1.0 identified two with twelve false positives, while the random forest model at the 0.5 threshold identified one with none. Thirty of the 121 test patients (24.8%) fell in the FIB-4 indeterminate range (1.30–2.67); the model’s performance within that subgroup was not analysed separately and is not claimed. This comparison concerns the re-analysis cohort and does not reproduce the initial 406-record cirrhosis experiment (Section 4.6). On these data, a machine learning model trained on the 12 available predictors does not outperform FIB-4 for histological cirrhosis, and the appropriate role of the analytic core for that stage is to present FIB-4 and APRI alongside a model estimate rather than to replace them.

### 4.6. Archived Development-Phase Estimates and Provenance Audit

The original submission reported ROC-AUC values of 0.90, 0.98 and 0.92 for the MASLD, cirrhosis and HCC modules, together with an ablation study and text-quality metrics for the language layer. Before revision, the archived model, imputer and scaler files, the derivative data tables and the application code were audited to establish what each value rests on (Table 8). HCC module: The saved SVM and scaler correspond to a 163-record training partition drawn by a stratified 80:20 split (random_state 42) from a 204-record table that had already been class-balanced from the 165 source patients. Re-scoring the saved model on the reconstructed 41-record test partition reproduces an ROC-AUC of 0.924 (accuracy 0.805, Brier 0.136). Because balancing preceded the split, duplicated minority records may occur in both partitions, and the value is retained only as an archived, optimistic development estimate of one-year survival discrimination, not as leakage-controlled validation. MASLD module: The archived result figure documents a 21-record evaluation with an ROC-AUC of 0.90 and accuracy 0.857, and the saved CatBoost model documents an 81-record fit; the saved scaler, however, was fitted on all 605 records, and neither the identities of the 21 test patients nor the intermediate sampling table were retained. The value is therefore reported as an archived development estimate that cannot be reconstructed or paired with calibration or decision-curve analysis. Cirrhosis module; The retained training output records AUC 0.979179, accuracy 0.926829 and F1 0.925 on 82 test records, with 41 records per class, drawn from a balanced table of 406 records. The value of 0.98 is therefore retained as an initial development estimate. The source tables and individual test probabilities are unavailable, and correspondence between that run and the saved binary remains unresolved (File S5). This experiment cannot be identified as the 605-patient F4 analysis. Language layer. The recovered 4104-record corpus and training log document adaptation, but raw evaluation answers, query-level metric outputs and rater records were not retained. Previously reported language-quality and expert-rating values are not used as verified results. The retained run has aggregate training loss 0.04984 over 1285 steps; the last logged loss is 0.022695 at step 1280. These are optimisation measurements, not held-out language-quality results (File S4).

### 4.7. Interoperability Design and Evaluation Readiness

For the results above to be reproducible outside the development environment, the system must be able to receive data from, and report back into, the systems a hospital already runs.

All eleven fields of the input schema were mapped to LOINC codes and all three disease categories to ICD-10, defining how laboratory data with different local names could resolve to the same feature (Table 4). On the output side, the three artefacts the system produces are expressed as HL7 FHIR R4 resources rather than as a proprietary payload: laboratory inputs as Observation, stage-specific risks as RiskAssessment, and the guideline-grounded narrative as DiagnosticReport (Table 9). These mappings provide an implementation specification for future reference-server and site-level testing; interoperability with a hospital information system was not demonstrated in this study.

Resources are exchanged as JavaScript Object Notation over RESTful endpoints and processed asynchronously. Formal FHIR conformance testing against a reference server was not performed, and this is stated as a limitation in Section 5.6.

Three further prototype properties are relevant to future site evaluation. First, data can enter through three channels (manual entry, optical character recognition of laboratory reports and voice input), reducing dependence on an existing structured feed (Figure 5). Second, the proposed prototype configuration specifies AES-256 at rest, TLS 1.3 in transit, OAuth 2.0 with role-based authorisation, and an immutable audit log of every prediction request; these controls have not undergone an independent security audit or a site-level information-governance review. Third, end-to-end response time was observed to be within a single consultation turn in the development environment, but it was not measured under a documented protocol and no latency figure is reported.

No site has yet run HepDecide against live data, no conformance certificate has been obtained, and no clinician has used it in a real consultation. The claim made here is one of readiness for evaluation, not of demonstrated implementation. Isolated numerical checks found a platelet-unit inconsistency in the archived APRI and HCC score methods. With AST 40 U/L and platelets 200 × 10^9^/L, reference APRI is 0.5, whereas the archived cirrhosis method returns 500. The re-analysis used separately implemented reference formulae. These checks and their scope are reported in File S6.

### 4.8. Illustrative Case Studies

**Figure 12:**
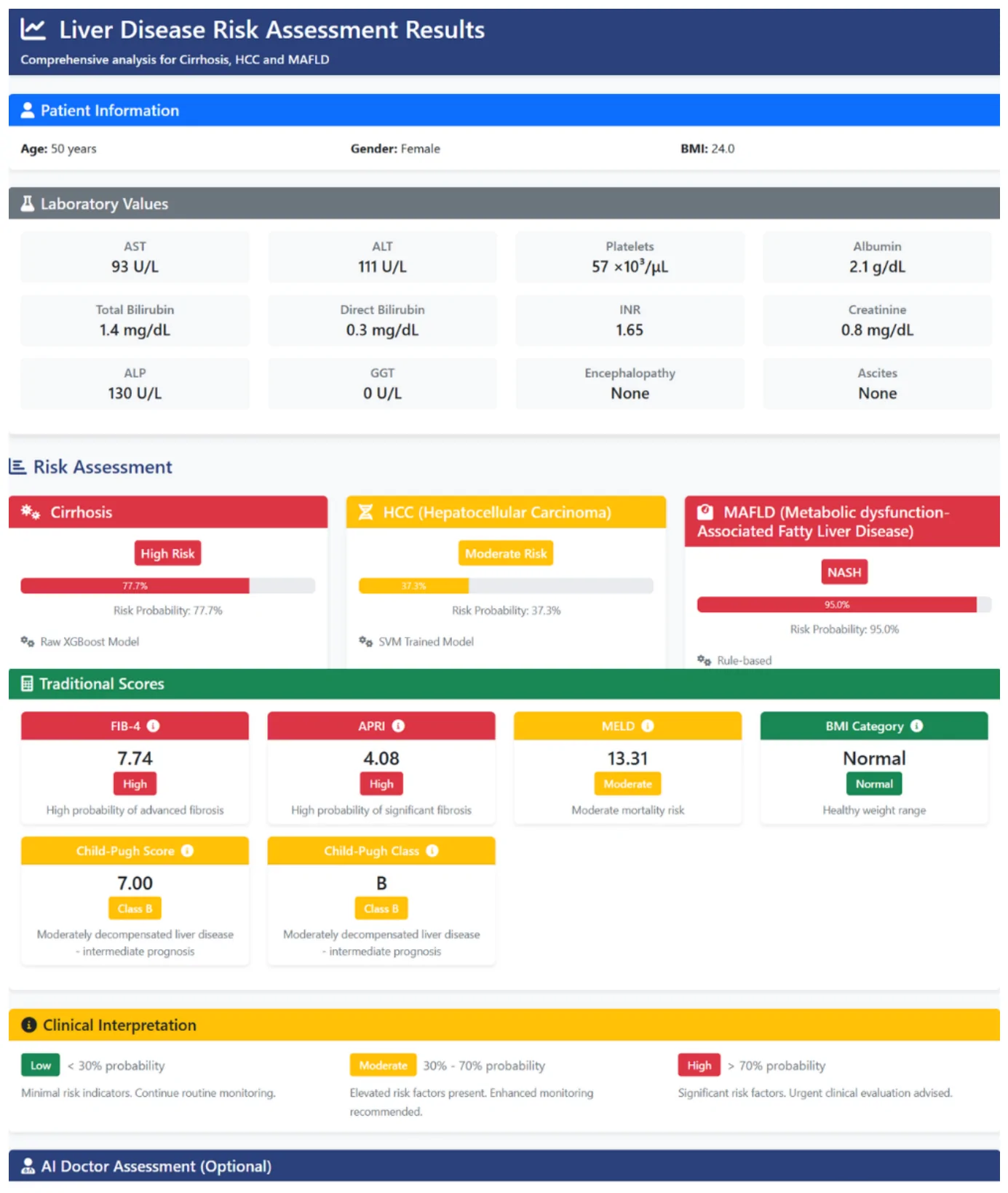
HepDecide interface—Case 1: multi-pathology, high-risk profile with concordant traditional scores (prototype output, illustrative).

**Figure 13:**
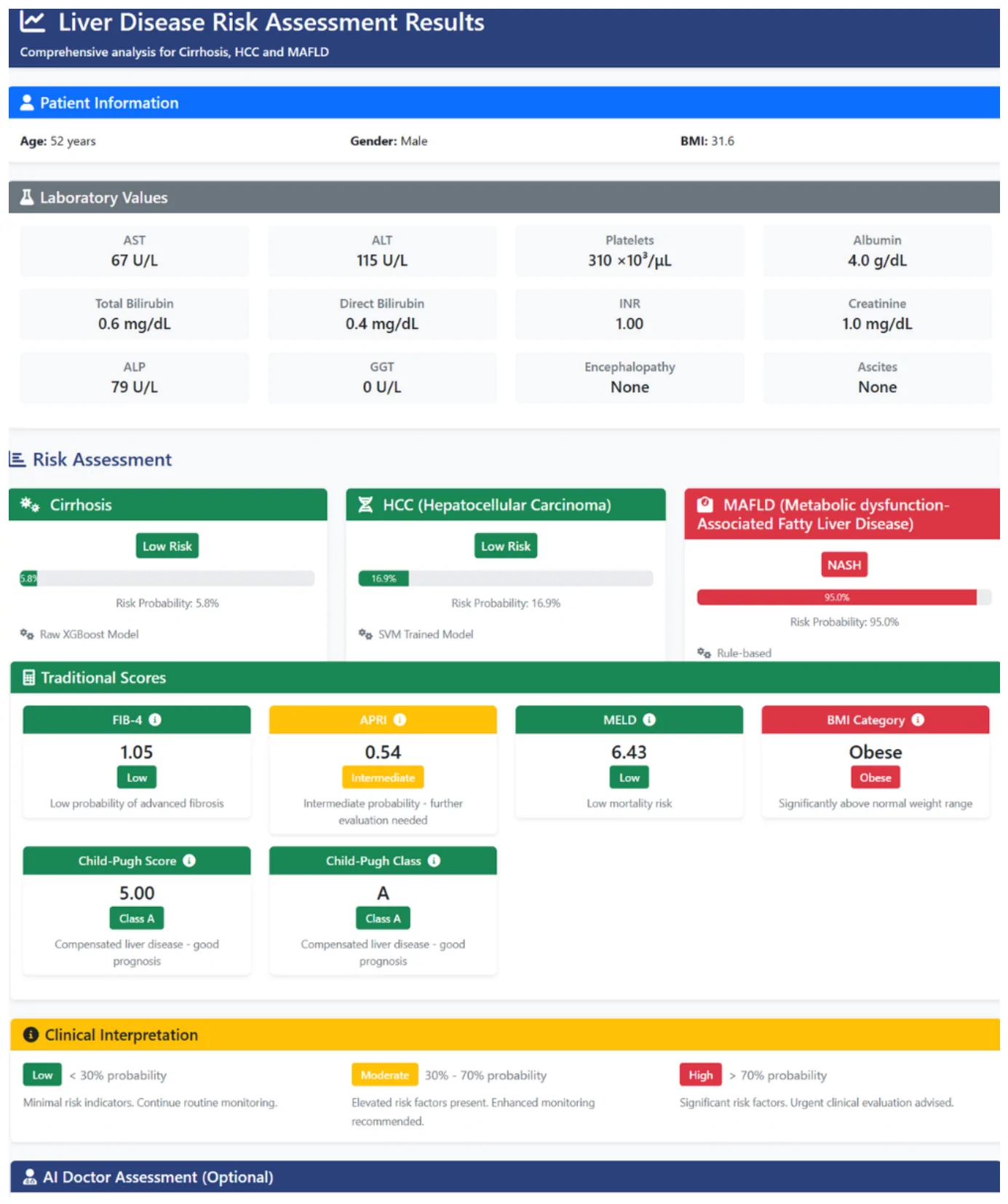
HepDecide interface—Case 2: obese patient with normal biochemistry displayed as low cirrhosis/HCC-severity score (prototype output, illustrative).

To illustrate how HepDecide’s risk scores and guideline-grounded reports are presented to a clinician, we describe two retrospective, de-identified scenarios as displayed by the prototype interface (Figure 12 and Figure 13). The risk values shown were produced by the development-phase prototype models, whose development estimates are described in Section 4.6; the cases therefore illustrate the interface and the way scores, traditional indices and explanations are assembled in one view, and are not evidence of accuracy.

#### 4.8.1. Case 1—Advanced Injury with Fibrosis Concordance

A 50-year-old woman presented with elevated liver enzymes and severe thrombocytopenia (platelets 57 × 10^3^/μL). The stage-specific expert models estimated a cirrhosis probability of 77.7% and a steatohepatitis (MASLD-stage) probability of 95%. These estimates were concordant with the patient’s traditional indices (FIB-4 = 7.74; APRI = 4.08; Child–Pugh class B); the displayed calculations were not independently validated. The SHAP analysis attributed the dominant contribution to the low platelet count, mirroring the clinical reasoning that thrombocytopenia reflects portal hypertension in advanced disease (Figure 12).

#### 4.8.2. Case 2—Biochemical Pattern Recognition and False-Positive Avoidance

A 52-year-old man with a body-mass index of 31.6 kg/m^2^ (obese) but otherwise normal laboratory values was assessed. Despite physical obesity, the prototype displayed low cirrhosis and HCC-severity scores (5.8% and 16.9%, respectively; Child–Pugh class A), reflecting the healthy underlying biochemical pattern despite an elevated metabolic score. This case illustrates the interface layout, not a measured reduction in false positives (Figure 13). The HCC-labelled output cannot be interpreted as HCC-detection probability because the source endpoint is survival among patients with diagnosed HCC.

Together, these scenarios illustrate the intended behaviour of the interface at both ends of the risk spectrum: concordance of a model estimate with the traditional scores in a high-risk profile, and a low score in a metabolically unhealthy but biochemically normal patient. Whether the prototype behaves this way systematically is a question for the evaluation in Section 4.1, Section 4.2, Section 4.3, Section 4.4 and Section 4.5 and for prospective testing, not for case illustrations.

## 5. Discussion

### 5.1. Principal Findings

This study set out to close two persistent gaps in hepatology decision support: the fragmentation of static laboratory scores across the clinical workflow, and the disconnection between quantitative model outputs and the natural-language reasoning clinicians apply at the bedside. HepDecide addresses both by coupling stage-specific machine learning models with a SHAP-based explainability layer and a guideline-grounded, retrieval-augmented language model inside a standards-oriented pipeline. The principal empirical finding of the revised evaluation is sobering. Under a leakage-controlled protocol on the same public cohorts, the predictive layer showed moderate discrimination (ROC-AUC 0.67, 0.81 and 0.76 for steatohepatitis, cirrhosis and one-year HCC survival) with wide confidence intervals, the cirrhosis model did not outperform FIB-4 on the same test partition, and the steatohepatitis model added little over the base rate at a fixed threshold. The archived development-phase estimates were higher, but the effects of different data derivatives, partitions, preprocessing and model selection cannot be separated from the available records. The retained cirrhosis training output documents the initial AUC of 0.98; it does not supply the original test probabilities. The language layer, whose grounding is the component most likely to matter for safety, could not be evaluated from the retained records. These retrospective findings therefore support the design rationale of the system and the transparency of its evaluation pipeline, but they do not support a claim of predictive superiority over existing indices, and they underline that prospective external and workflow evaluation is required to establish any clinical benefit.

### 5.2. What the Re-Analysis Does and Does Not Show

Three points follow from the re-analysis. First, discrimination for histological cirrhosis was driven overwhelmingly by platelet count (Figure 11b), which is also a component of FIB-4 and APRI; the model uses 12 available predictors, but the small number of F4 cases limits conclusions about added predictive value. Consistent with reports that machine learning models outperform static indices only when trained on substantially larger cohorts [13,14,15,16,43,44], the present data are too small to show such an advantage, and the confidence intervals say so. Second, the steatohepatitis endpoint is difficult by construction: 88.8% of the biopsy cohort is positive, the negative class has 68 patients, and the routine chemistry available here separates the two histological diagnoses only weakly (ROC-AUC 0.67), which is in line with the known limitations of non-invasive markers for steatohepatitis as opposed to fibrosis [5,6]. Third, the sensitivity analyses show that neither the imputation method nor the choice among class-weighting, random over-sampling and no correction changes the conclusions (Table 6); random under-sampling was consistently harmful, in keeping with simulation evidence that imbalance corrections rarely help and can hurt calibration [25]. The re-analysis was not preregistered and its model-selection rule was fixed before the test set was scored; it should be read as a transparent internal evaluation, not as an external validation.

### 5.3. The Language-Generation Layer

Unconstrained LLMs frequently produce plausible but unsupported clinical statements, including incorrect drug-dosing information [28,29], and retrieval grounding is the most widely adopted mitigation for this failure mode [30,31,32,48]. HepDecide implements that mitigation, and its design places the retrieved guideline passages, the stage-specific risk, the SHAP attributions and the biochemical profile in a single structured prompt so that every narrative statement can in principle be traced to a source. Whether the implementation achieves the reductions in unsupported content reported in the literature was measured during development, but the query-level outputs and rater records were not retained and the corresponding results have been withdrawn (Section 4.6). No claim about the text quality, hallucination rate or clinician acceptance of the generated narrative is therefore made here; the retrieval configuration actually released (Section 3.6) also differs from the design target in corpus scope and passage count, and both must be evaluated before such a claim can be supported. The architectural point stands regardless of that evaluation: in an LLM-assisted CDSS, procurement and governance attention belongs on the retrieval and provenance mechanism as much as on classifier accuracy, because the narrative is what the clinician reads.

### 5.4. Implications for Prospective Workflow Evaluation

Feature-level LOINC mapping, ICD-10 alignment, and an HL7 FHIR R4 resource model specify how heterogeneous hospital data and results could be exchanged without loss of semantic integrity [33,34,53]. Response time in the development environment fell within a single consultation turn but was not measured under a documented protocol, so no latency figure is reported and workflow compatibility remains to be tested. The explainability layer converts SHAP attributions into clinician-readable rationale [19,20,21], addressing one component of the black-box barrier while not, by itself, demonstrating clinical usefulness [22].

It follows from the evidence presented that the appropriate claim for this system is a bounded one. On locked test partitions of 121, 121 and 33 patients, with five cirrhosis events and confidence intervals as wide as 0.52–1.00, and without an advantage over FIB-4, HepDecide is intended as a clinician-supervised triage and reporting prototype whose proposed effect on non-specialist referral decisions remains to be tested prospectively. It is not appropriate, on this evidence, for it to act as a determinant of clinical management: every output is presented to a clinician for acceptance rather than acted upon automatically, the traditional indices are displayed alongside the model estimate, and the proposed audit log would record who saw which recommendation and when.

### 5.5. Requirements for Prospective Implementation

Translating these results into service use raises questions that are organisational rather than computational. On data governance, the technical controls described in Section 4.7 map onto what a hospital information-governance review normally asks for, and the proposed immutable audit log would provide the record that such a review requires. What is missing is a site-level data-protection impact assessment, which cannot be written in the abstract because it depends on the deploying institution’s legal basis for processing. On accountability, the system is positioned as a clinician-facing aid whose outputs require acceptance, which places responsibility for the decision with the treating clinician. This is the position that emerging regulatory frameworks for clinical decision support take, and the interface is intended to support clinician oversight.

On oversight, the guideline-grounded generation is bounded by the currency of the indexed corpus, which means a deploying service inherits a maintenance obligation: when EASL or AASLD issue revised guidance, the vector index must be rebuilt or the system will continue to cite superseded recommendations with full confidence. This is a foreseeable failure mode of any retrieval-grounded clinical tool and it needs an owner at the deploying site. On workforce implications, the intended effect is to redistribute rather than replace effort, moving the assembly of fragmented scores off the clinician while leaving interpretation with them. Whether that effect is realised is precisely what a prospective workflow study would have to measure, and this study does not measure it.

### 5.6. Limitations and Generalisability

Several limitations should be acknowledged. First, the models were evaluated internally on locked test partitions of the development cohorts and have not been tested on a prospective, independent, multi-centre external cohort; the calibration and decision-curve analyses reported here are internal and exploratory, and external validation with pre-specified thresholds is required before any clinical use [23,24,25]. Second, the event counts are small (five F4 patients and 14 simple-steatosis patients in the respective test partitions; 33 patients in the HCC test partition), which is the direct cause of the wide confidence intervals; the source cohorts are specialist referral series whose disease distribution differs from primary care, and the HCC cohort contains no HCC-negative controls (Section 3.2). Third, recruitment settings and periods are incompletely documented and the HCC cohort is 90% cirrhotic, so any between-stage comparison partly reflects inter-cohort distributional differences rather than biological stage separation; public availability facilitates reproducibility at the source-data level, but source-specific endpoint definitions and missingness patterns may affect replication, and source-stratified replication is needed. Fourth, preprocessing and balancing differed across the initial experiments (Section 3.3), with some steps preceding partitioning. The retained cirrhosis output documents AUC 0.979179, but its original input tables and individual test probabilities are unavailable, preventing independent recalculation on the original test set. Patient overlap and correspondence to the saved binary remain unresolved. Fifth, the language-generation layer, its QLoRA adaptation and its retrieval configuration are described but not evaluated, because raw evaluation answers, query-level metrics and rater records were not retained; the recovered corpus and training log document adaptation but not clinical performance; the blinded rating exercise conducted during development involved three hepatologists whose institutions, seniority and recruitment were also not documented, and no result from it is reported. Sixth, no clinician has used the system in a live consultation, no site has run it against live data, no latency was measured under a documented protocol, and formal FHIR conformance testing and an independent security review were not performed, so the interoperability properties reported in Section 4.7 constitute readiness for evaluation rather than demonstrated implementation. Finally, the system is intended to augment rather than replace clinical judgement: its outputs require physician oversight, and the guideline-grounded generation is bounded by the coverage and currency of the indexed guidelines. HCC source platelet scales are inconsistent, and demographic subgroup performance was not assessed.

### 5.7. Future Directions

Several elements of the pipeline were implemented during development but are not evaluated here and are the subject of future work. First, the interpretation layer was domain-adapted by QLoRA on a curated question–answer corpus; although the corpus and training log were recovered, annotation and clinical quality-control records remain unavailable and no validation split was defined, a documented and pre-registered fine-tuning protocol with a held-out evaluation set is required before any text-quality claim can be made for that component. Second, the retrieval layer (BAAI/bge-m3 embeddings, 800-character chunks with 100-character overlap, cosine similarity, top-2 passages, 36 PDFs and three text files in the repository) has not been evaluated for retrieval precision or latency, and neither re-ranking nor restriction of the corpus to the EASL and AASLD guidelines has been tested; faithfulness (RAGAS), factual precision (FactScore) and semantic similarity (BERTScore) will be reported with query-level records retained [71,72,73]. Third, the exploratory blinded report-rating exercise conducted during development did not retain rater records; a larger, multi-institutional rater study using a published rating instrument [51,52], with pre-specified rater characteristics and blinded randomised presentation, is planned. Fourth, the decision curves reported in Section 4.3 are exploratory; the step that would change what can be claimed is a prospective evaluation in a live service with pre-specified clinical thresholds, a defined care pathway, a defined user group, and measured effects on referral decisions and on the time clinicians spend assembling risk information. Alongside these, prospective multi-centre external validation, integration of elastography and imaging features, assessment of algorithmic fairness across demographic subgroups [77], privacy-preserving federated training [78], formal FHIR conformance testing, a documented latency protocol and an independent security review remain necessary before any clinical use. Because predictive performance is known to degrade between development and deployment [79], a full FHIR-based deployment with prospective monitoring will be required to measure real-world impact.

## 6. Conclusions

HepDecide is a modular, explainable, and guideline-grounded clinical decision support prototype for the chronic liver-disease spectrum that unifies stage-specific expert modelling, SHAP-based explainability, and retrieval-augmented language generation within a single, standards-oriented pipeline. In a leakage-controlled retrospective re-analysis on public cohorts, its predictive layer showed moderate discrimination with wide confidence intervals (ROC-AUC 0.67–0.81), Brier scores of 0.096, 0.028 and 0.199, and no advantage over FIB-4 for histological cirrhosis; higher development-phase estimates of 0.90, 0.98 and 0.92 are reported separately, with the available evidence and preprocessing limitations in Section 4.6. The language-generation layer is implemented but not evaluated from the retained records. Practically, the prototype presents a stage-specific risk estimate, the biomarkers that drove it, the traditional indices, and a guideline-traceable narrative in one view, and its LOINC, ICD-10 and FHIR R4 mappings provide a standards-oriented starting point for formal conformance and prospective site-integration testing. Two implications follow. First, for small specialist cohorts of this size, a machine learning layer over routine chemistry should be positioned as a companion to FIB-4 and APRI rather than a replacement, and every reported estimate should be accompanied by its provenance and its confidence interval. Second, the grounding layer requires the same scrutiny as the classifier, and that scrutiny has yet to be applied here. Until external, text-quality and live-workflow evaluations are completed, HepDecide should be regarded as a retrospectively evaluated prototype intended for clinician-supervised triage and reporting, not as a clinically ready system or a substitute for specialist assessment.

## Figures and Tables

**Table 1:** Closest system-level comparators and the boundary of HepDecide’s contribution.

System and Scope	Prediction and Explanation	Guideline or Language Layer	Interoperability and Evaluation Boundary
Choi et al. [38], HCC treatment	ML treatment recommendation and survival prediction; no patient-level explanation layer	No RAG or guideline-grounded narrative	Single-centre retrospective development and validation; no FHIR layer
Kresevic et al. [39], chronic HCV guidance	No patient-level predictive classifier or SHAP layer	Hepatology guideline interpretation with RAG and expert assessment	Guideline-delivery study; no reported FHIR implementation
GastroBot [40], gastrointestinal question answering	No patient-specific risk classifier or SHAP layer	RAG over gastrointestinal guidelines and articles	RAGAS and manual question evaluation; no liver-stage workflow or FHIR layer
FibroAgent [41], MASLD advanced fibrosis	XGBoost prediction with patient-level SHAP explanations	Risk pathways, education, and structured reports; no evaluated guideline-RAG layer	Proof-of-concept scenarios; single fibrosis task and no FHIR layer
FHIR-RAG-MEDS [42], four non-liver conditions	No disease-specific predictive classifier or SHAP layer	Patient-specific, guideline-grounded RAG	SMART on FHIR with physician-question evaluation; not a liver-stage prediction system
HepDecide, chronic liver spectrum	Stage-specific models with patient-level SHAP attribution	EASL/AASLD-grounded narrative generation	FHIR R4-oriented outputs; leakage-controlled internal retrospective evaluation of the predictive layer; no formal conformance, live deployment or evaluated language layer

**Table 2:** Clinical characteristics of the study cohorts (mean ± SD).

Parameter	Reference Range	MASLD Cohort [58] (*n* = 605)	Cirrhosis Subgroup (F4) [58] (*n* = 24)	HCC Cohort [59] (*n* = 165)
Age (years)	–	46.3 ± 10.6	55.2 ± 9.1	64.7 ± 13.3
AST (U/L)	5–40	48.7 ± 30.2	54.7 ± 32.5	96.4 ± 87.5
ALT (U/L)	7–56	76.5 ± 49.4	56.9 ± 34.5	67.1 ± 57.5
ALP (U/L)	40–130	108.8 ± 66.8	106.4 ± 62.3	212.2 ± 167.9
GGT (U/L)	8–61	69.1 ± 68.7	97.5 ± 56.3	268.0 ± 258.8
Albumin (g/dL; HCC recorded scale *)	3.5–5.0	4.67 ± 0.38	4.42 ± 0.48	3.45 ± 0.69
Total bilirubin (mg/dL)	0.1–1.2	0.80 ± 0.60	0.82 ± 0.38	3.09 ± 5.50
Direct bilirubin (mg/dL)	0.0–0.3	0.21 ± 0.17	0.21 ± 0.13	1.93 ± 4.21
Platelets (10^3^/μL)	150–450	241.9 ± 68.0	160.9 ± 38.7	— *
INR	0.8–1.2	1.04 ± 0.16	1.10 ± 0.11	1.42 ± 0.48
Creatinine (mg/dL)	0.6–1.2	0.82 ± 0.20	0.71 ± 0.20	1.13 ± 0.96
Body-mass index (kg/m^2^)	–	31.9 ± 5.1	35.1 ± 4.5	—

* In the HCC source file, platelets are recorded under a single G/L label but with values ranging from 1.71 to 459,000, so no common-unit mean is reported; albumin is shown on the recorded numerical scale because the repository unit label is inconsistent (Section 3.2). A dash marks a variable not tabulated for the cohort concerned.

**Table 3:** Cohort composition and class distribution before resampling. Counts are those of the source datasets as released; the resampling described in Section 3.4 was applied only within training data after these counts were taken.

Characteristic	MASLD Cohort [58] (*n* = 605)	HCC Cohort [59] (*n* = 165)
Sex, female/male	284 (46.9%)/321 (53.1%)	32 (19.4%)/133 (80.6%)
Age, years, mean ± SD	46.3 ± 10.6	64.7 ± 13.3
Diabetes mellitus	225 (37.2%)	56/162 (34.6%); 3 missing
Hypertension	214 (35.4%)	—
Metabolic syndrome	392 (64.8%)	—
Cirrhosis present	24 (4.0%)	149 (90.3%)
MASLD-stage label (SAF): steatohepatitis/simple steatosis	537 (88.8%)/68 (11.2%)	—
Fibrosis stage (Kleiner): F0/F1/F2/F3/F4	196 (32.4%)/190 (31.4%)/111 (18.3%)/84 (13.9%)/24 (4.0%)	—
Any fibrosis (F1–F4)	409 (67.6%)	—
Cirrhosis-stage label: F4/non-F4	24 (4.0%)/581 (96.0%)	—
HCC-stage label: died within 1 year/survived	—	63 (38.2%)/102 (61.8%)
Missing values	INR and creatinine recorded in 304/305 patients; all other schema fields ≥ 99.5% complete	10.2% overall across 49 source fields; 8 patients (4.8%) complete

**Table 4:** The eleven-field canonical input schema of HepDecide, with LOINC codes, units, and clinical significance. Alpha-fetoprotein is populated only by the HCC cohort; creatinine and INR are populated for approximately half of the MASLD cohort (see Section 3.2).

Clinical Parameter	LOINC	Unit	Clinical Significance
Age	30525-0	years	Demographic risk
Aspartate aminotransferase (AST)	1920-8	U/L	Hepatocellular damage
Alanine aminotransferase (ALT)	1742-6	U/L	Hepatocellular damage
Albumin	1751-7	g/dL	Synthetic function
Total bilirubin	1975-2	mg/dL	Cholestasis/excretion
Platelets	777-3	10^3^/μL	Portal hypertension
Alkaline phosphatase (ALP)	6768-6	U/L	Cholestatic injury
Gamma-glutamyl transferase (GGT)	2324-2	U/L	Biliary dysfunction
Creatinine	2160-0	mg/dL	Renal function (MELD)
International normalised ratio (INR)	6301-6	ratio	Coagulation/synthesis
Alpha-fetoprotein (AFP)	1834-1	ng/mL	HCC tumour marker

**Table 5:** Performance of the selected configuration for each endpoint on its locked test partition in the leakage-controlled re-analysis (calibrated probabilities). The 95% confidence intervals are percentile bootstrap intervals over the test set. Sensitivity and specificity are at the fixed 0.5 probability threshold. CV ROC-AUC is the mean ± SD of five-fold cross-validation on the training set for the selected configuration.

Endpoint	Test *n* (Events)	Selected Configuration	CV ROC-AUC	Test ROC-AUC (95% CI)	Average Precision (95% CI)	Brier (95% CI)	Sens./Spec. at 0.5
Steatohepatitis vs simple steatosis	121 (107)	CatBoost, class weighting	0.76 ± 0.07	0.67 (0.50–0.84)	0.93 (0.88–0.98)	0.096 (0.056–0.138)	1.00/0.00
Histological cirrhosis (F4) vs F0–F3	121 (5)	Random forest, random over-sampling	0.91 ± 0.06	0.81 (0.52–1.00)	0.56 (0.05–1.00)	0.028 (0.008–0.056)	0.20/1.00
One-year survival in diagnosed HCC	33 (20)	Random forest, random over-sampling	0.80 ± 0.07	0.76 (0.56–0.92)	0.79 (0.60–0.97)	0.199 (0.135–0.269)	0.95/0.31

**Table 6:** Sensitivity analyses in the re-analysis. Left: locked test performance of the selected configuration under KNN (primary) and median imputation, both fitted on training data only. Right: mean five-fold cross-validated ROC-AUC on the training set for the selected classifier under each class-imbalance strategy; the selected strategy is marked with an asterisk.

Endpoint	KNN: ROC-AUC/Brier	Median: ROC-AUC/Brier	None	Class Weight	Random Over	Random Under	SMOTE
Steatohepatitis (CatBoost)	0.674/0.096	0.698/0.091	0.739	0.759 *	0.743	0.704	0.728
Cirrhosis F4 (random forest)	0.814/0.028	0.791/0.030	0.907	0.909	0.911 *	0.870	0.897
HCC one-year survival (random forest)	0.762/0.199	0.796/0.189	0.786	0.790	0.797 *	0.769	0.785

**Table 7:** Cirrhosis (F4) identification on the locked 121-patient test partition of the re-analysis: FIB-4 and APRI at their conventional thresholds versus the selected random forest model at the 0.5 probability threshold. ROC-AUC confidence intervals are percentile bootstrap intervals.

Score/Model	ROC-AUC (95% CI)	Average Precision	TN/FP/FN/TP	Accuracy	F1-Score
FIB-4 (>3.25)	0.92 (0.82–0.99)	0.39	114/2/3/2	0.96	0.44
APRI (>1.0)	0.83 (0.69–0.97)	0.20	104/12/3/2	0.88	0.21
Random forest, re-analysis (>0.5)	0.81 (0.52–1.00)	0.56	116/0/4/1	0.97	0.33

**Table 8:** Provenance audit of the archived development-phase estimates. “Retained” denotes an archived estimate reported for transparency only; no calibration, decision-curve or SHAP result in this paper is attributed to an archived model.

Module (Archived Value)	What the Archived Artefacts Show	Status in This Version
HCC, ROC-AUC 0.92	Saved SVM and scaler match a 163/41 stratified split of a 204-record table balanced before splitting; 0.924 reproduced on the 41-record partition; endpoint is one-year survival	Retained as an archived, optimistic development estimate
MASLD, ROC-AUC 0.90	Archived figure documents a 21-record evaluation; model fit on 81 records; scaler fitted on all 605 records; test identities not retained	Retained as an archived development estimate; not reconstructable
Cirrhosis, ROC-AUC 0.98	Retained training output: AUC 0.979179, accuracy 0.926829, F1 0.925; 82 test records (41 per class) from a balanced table of 406 records. Original tables and individual predictions unavailable.	Retained as an initial development estimate; exact saved-model correspondence unresolved
Ablation, text-quality metrics, expert ratings, latency	Corpus and training log recovered; raw evaluation answers, query-level metrics and rater records unavailable	Training methods and loss reported in File S4; language-quality claims not verified

**Table 9:** Mapping between HepDecide outputs and HL7 FHIR R4 resources, with the terminology binding used for each.

HepDecide Artefact	FHIR R4 Resource	Terminology Binding	Direction
Laboratory value (11 parameters)	Observation	LOINC	Inbound and outbound
Stage-specific risk probability	RiskAssessment	ICD-10 (condition)	Outbound
Guideline-grounded clinical narrative	DiagnosticReport	ICD-10 (condition)	Outbound
SHAP attribution set (top five)	Observation (component)	LOINC (source parameter)	Outbound

## Data Availability

The two source datasets used for the re-analysis are openly available. The biopsy-proven MASLD cohort is available at https://www.kaggle.com/datasets/tourdeglobe/fatty-liver-disease (accessed on 11 August 2026) [58], and the hepatocellular carcinoma cohort is the UCI Hepatocellular Carcinoma Survival dataset, available at https://archive.ics.uci.edu/dataset/423/hcc+survival (accessed on 11 August 2026) [59]. No new patients were recruited; derived analysis outputs are supplied. The re-analysis code and outputs and the archived development-phase model files are provided as Files S2 and S3; the development-phase application code is available at https://github.com/fatmanurakbas/bitirme_calismasi (accessed on 11 August 2026). Files S4–S6 supply the additional methods and outputs. The original cirrhosis input tables and individual test probabilities are unavailable; the language-model checkpoint, saved retrieval index and raw language-evaluation records were not supplied.

## Supplementary Materials

The following supporting information can be downloaded at https://www.mdpi.com/article/10.3390/healthcare14203397/s1, File S1: Completed TRIPOD + AI reporting checklist. File S2: Re-analysis code, unit tests, pinned requirements, SHA-256 hashes of the source files, run manifest, per-endpoint benchmark tables (25 configurations each), imputation-sensitivity tables, patient-level locked test predictions, and all figure-generating outputs. File S3: Provenance audit of the archived development-phase model files (audit report, machine-readable audit record and reconstructed HCC test predictions). File S4: Language-model training and retrieval evidence, including the retained loss curve. File S5: Initial experiment methods and available records. File S6: All-algorithm diagnostic plots, SHAP arrays, reference baselines, nested cirrhosis analysis and numerical implementation checks.

## Abbreviations

The following abbreviations are used in this manuscript:

AASLD	American Association for the Study of Liver Diseases
AFP	Alpha-fetoprotein
ALP	Alkaline phosphatase
ALT	Alanine aminotransferase
AP	Average precision
APRI	Aspartate aminotransferase-to-platelet ratio index
AST	Aspartate aminotransferase
CDSS	Clinical decision support system
CI	Confidence interval
DCA	Decision-curve analysis
EASL	European Association for the Study of the Liver
EHR	Electronic health record
FAISS	Facebook AI Similarity Search
FHIR	Fast Healthcare Interoperability Resources
FIB-4	Fibrosis-4 index
GGT	Gamma-glutamyl transferase
HCC	Hepatocellular carcinoma
HIPAA	Health Insurance Portability and Accountability Act
HL7	Health Level Seven
ICD-10	International Classification of Diseases, 10th revision
INR	International normalised ratio
JSON	JavaScript Object Notation
KNN	k-nearest neighbours
KVKK	Turkish Personal Data Protection Law
LLM	Large language model
LOINC	Logical Observation Identifiers, Names and Codes
LoRA	Low-rank adaptation
MASLD	Metabolic dysfunction-associated steatotic liver disease
MELD	Model for End-Stage Liver Disease
ML	Machine learning
OCR	Optical character recognition
QLoRA	Quantized low-rank adaptation
RAG	Retrieval-augmented generation
RAGAS	Retrieval-augmented generation assessment
ROC-AUC	Area under the receiver operating characteristic curve
SHAP	SHapley Additive exPlanations
SMOTE	Synthetic minority over-sampling technique
SVM	Support vector machine
TRIPOD + AI	Transparent Reporting of a multivariable prediction model for Individual Prognosis Or Diagnosis + Artificial Intelligence
XAI	Explainable artificial intelligence
