# Supplementary material for: HepDecide: A Standards-Oriented, Explainable Clinical Decision Support Prototype for the Chronic Liver-Disease Spectrum—Design, Guideline-Grounded Reporting, and a Leakage-Controlled Retrospective Evaluation
Source: Healthcare (Basel). 2026 Oct 12;14(20):3397. doi: 10.3390/healthcare14203397 (PMC13704185; doi:10.3390/healthcare14203397)
Supplement: Supplementary file 1 [file healthcare-14-03397-s001.zip › File S1_TRIPOD_AI_Reporting_Map.pdf]

Supplementary File S1. TRIPOD+AI Reporting Map

Item numbers follow TRIPOD+AI (2024) [manuscript reference 52]. The table records the current reporting status; it is not a declaration that every methodological requirement has been met. Locations are section/table identifiers in the clean manuscript and supplements, which remain stable across Word pagination. Item descriptions are abbreviated.

Item	Topic	Reporting status / available information	Location
1	Title	Reported	Title
2	Abstract	Reported with limits	Abstract
3a	Clinical context and rationale	Reported	Introduction; Table 1
3b	Target population, users and intended use	Reported; clinical effect not evaluated	Introduction; Sections 3.2, 5.4
3c	Health inequalities	Not characterised in the source populations	Section 5.6
4	Objectives	Reported	Introduction; Section 3.1
5a	Data sources and representativeness	Reported for Group B; initial derivative composition incomplete	Section 3.2; S5
5b	Recruitment and follow-up dates	Not documented sufficiently in supplied source records	Section 3.2
6a	Setting and centres	Portuguese university hospital for HCC; biopsy-cohort institution unavailable	Section 3.2
6b	Eligibility	All source records; no additional restrictions	Section 3.2
6c	Treatments	Treatment adjustment not possible from documented analysis inputs	Section 3.2
7	Data preparation and quality checks	Reported; source-unit limitations retained	Section 3.3; S2, S6
8a	Outcome definition and time horizon	SAF diagnosis; F4; one-year HCC survival	Section 3.2; Table 3
8b	Outcome assessors	Qualifications and demographic information unavailable	Section 3.2
8c	Outcome-assessment blinding	Not documented	Section 3.2
9a	Predictor selection	Fixed lists from available model interfaces; no histology-derived predictors	Section 3.2
9b	Predictor definitions and measurement	Lists and source scales reported; timing and unit issues unresolved	Section 3.2; Tables 2, 4; S2
9c	Predictor assessors	Measurement-personnel information unavailable	Section 3.2
10	Sample-size rationale	All available records; no formal calculation; small events acknowledged	Sections 3.2, 5.6
11	Missing data	KNN and median sensitivity; variable counts; initial-run timing	Sections 3.2–3.3, 4.2; S2, S5–S6

Item	Topic	Reporting status / available information	Location
12a	Partitioning and use of data	Stratified 80:20; nested 5 outer / 3 inner folds	Sections 3.8–3.9; S6
12b	Predictor handling	Within-training imputation; scaling for logistic and SVM; raw-unit KNN	Sections 3.3–3.4; S2
12c	Model development and internal evaluation	Five algorithms; fixed settings; 25 configurations per endpoint	Sections 3.4, 3.8–3.9; S2, S6
12d	Clustering and heterogeneity	No cluster identifiers or multi-centre evaluation available	Sections 3.2, 5.6
12e	Performance metrics and plots	AUC, AP, Brier, calibration, classification and exploratory DCA	Sections 3.8, 4.1–4.5; S6
12f	Model updating	Calibration described; no external model updating	Section 3.8
12g	Prediction calculation	Saved pipelines and sigmoid ensembles; probability threshold 0.5	Sections 3.4, 3.8; S6
13	Class imbalance	Five strategies; training-only resampling	Sections 3.4, 4.2; S2
14	Fairness	No subgroup performance or fairness intervention evaluated	Sections 3.8, 5.6–5.7
15	Model output and thresholds	Endpoint probabilities; fixed threshold; no clinical action threshold validated	Sections 3.8, 4.1, 4.3
16	Development/evaluation differences	Same-source holdouts; initial-run definitions differ; overlap unresolved	Sections 3.1–3.2, 3.9; S5
17	Ethics and consent	Source documentation limits; no waiver identifier supplied	Ethics and Consent statements
18a	Funding	Reported	Funding
18b	Conflicts	Reported	Conflicts of Interest
18c	Protocol access	Revision extension protocol retained; not original preregistration	Section 3.8; S6 protocol.json
18d	Registration	No registration identified in supplied records	Section 3.8
18e	Data access	Source data and derived outputs supplied; initial missing files stated	Data Availability; S2–S6
18f	Code access	Versioned scripts, dependencies, hashes and model files supplied	Data Availability; S2, S6
19	Patient/public involvement	Not documented in supplied study records	Section 3.1
20a	Record flow	605 → 484/121; 165 → 132/33; events provided	Sections 3.2, 3.8; S6, baseline_summary.csv
20b	Participant characteristics and missingness	Source-derived tables and variable-specific counts	Tables 2–3; S6
20c	Comparison of partitions	Counts/prevalence reported; no separate external cohort	S6, baseline_summary.csv
21	Sample and events per analysis	Holdout counts and 605/24 nested analysis reported	Table 5; S6
22	Model specification	Fixed settings, pipelines, binaries and predictions; initial identities incomplete	Sections 3.4, 4.6; S2–S6
23a	Performance and uncertainty	Holdout bootstrap / exact intervals; nested pooled results descriptive	Section 4.1; Table 5; S6

Item	Topic	Reporting status / available information	Location
23b	Performance by cluster	Not evaluated; cluster information unavailable	Section 5.6
24	Updated model results	Before/after calibration reported; no external updating	Section 4.1; S6
25	Interpretation and fairness	Internal estimates, baseline comparison and subgroup limits	Sections 5.1–5.4, 5.6
26	Limitations	Reported	Section 5.6
27a	Missing or poor-quality inputs in use	Imputation and source-unit issues reported; operational validation incomplete	Sections 3.3, 4.7; S6
27b	User interaction and expertise	Physician-facing prototype; no measured workflow benefit	Sections 3.7, 4.8, 5.4
27c	Further research	External, language, prospective workflow and technical validation	Section 5.7

Guideline: Collins et al. TRIPOD+AI statement. BMJ 2024;385:e078378. https://doi.org/10.1136/bmj-2023-078378. Official checklist: https://www.tripod-statement.org/wp-content/uploads/2024/04/TRIPODAI-Supplement.pdf
