# Supplementary material for: HepDecide: A Standards-Oriented, Explainable Clinical Decision Support Prototype for the Chronic Liver-Disease Spectrum—Design, Guideline-Grounded Reporting, and a Leakage-Controlled Retrospective Evaluation
Source: Healthcare (Basel). 2026 Oct 12;14(20):3397. doi: 10.3390/healthcare14203397 (PMC13704185; doi:10.3390/healthcare14203397)
Supplement: Supplementary file 1 [file healthcare-14-03397-s001.zip › File S2_Group_B_Data_and_Code.zip!/revision_analysis/source_data/hcc_survival.zip!/hcc-survival/hcc-description.txt]

Citation Request:
Please include this citation if you plan to use this database:

Miriam Seoane Santos, Pedro Henriques Abreu, Pedro J. García-Laencina, Adélia Simão, Armando Carvalho, “A new cluster-based oversampling method for improving survival prediction of hepatocellular carcinoma patients”, Journal of biomedical informatics, 58, 49-59, 2015.


1. Title: Hepatocellular Carcinoma Dataset (HCC dataset) 

2. Source Information
   -- Donors of database: 
	Miriam Seoane Santos (miriams@student.dei.uc.pt)
	Pedro Henriques Abreu (pha@dei.uc.pt)
	Department of Informatics Engineering, University of Coimbra, Portugal

	Armando Carvalho(aspcarvalho@gmail.com)
	Adélia Simão (adeliasimao@gmail.com) 
	Hospital and University Centre of Coimbra 

   -- Date: Feb, 2015
 

3. Past Usage:
Miriam Seoane Santos, Pedro Henriques Abreu, Pedro J. García-Laencina, Adélia Simão, Armando Carvalho, “A new cluster-based oversampling method for improving survival prediction of hepatocellular carcinoma patients”, Journal of biomedical informatics, 58, 49-59, 2015.

	-- Proposed a cluster-based oversampling approach robust to small and imbalanced datasets, accounting for the heterogeneity between HCC patients. The new approach 	is based on K-means clustering and a modification of SMOTE algorithm.
	
	-- The approach was coupled with NN and LR and compared to baseline approaches that do not consider clustering and/oversampling. 

	-- The target was the first-year survival of the patients, and the results were evaluated in terms of Accuracy, AUC 	values and F-measure.
 
	-- Data imputation was performed with KNN with the HEOM metric.

	-- The proposed approach (particularly, Augmented Sets Approach) coupled with NN presented better results regarding Accuracy (0.7519), AUC (0.7) and F-measure (0.6650).


4. Relevant Information:
HCC dataset was obtained at a University Hospital in Portugal and contais several demographic, risk factors, laboratory and overall survival features of 165 real patients diagnosed with HCC. The dataset contains 49 features selected according to the EASL-EORTC (European Association for the Study of the Liver - European Organisation for Research and Treatment of Cancer) Clinical Practice Guidelines, which are the current state-of-the-art on the management of HCC.

This is an heterogeneous dataset, with 23 quantitative variables, and 26 qualitative variables. Overall, missing data represents 10.22% of the whole dataset and only eight patients have complete information in all fields (4.85%). The target variables is the survival at 1 year, and was encoded as a binary 			variable: 0 (die) and 1 (lives). A certain degree of class-imbalance is also present (63 cases labeled as “dies” and 102 as “lives”).

A detailed description of the HCC dataset (feature’s type/scale, range, mean/mode and missing data percentages) is provided in Santos et al. “A new cluster-based oversampling method for improving survival prediction of hepatocellular carcinoma patients”, Journal of biomedical informatics, 58, 49-59, 2015.


5. Number of Instances: 165 

6. Number of Attributes: 49 + the class attribute


7. Attribute Information:
   
Name							Data Type		Abbreviation		Range/Possible Values		Missing Values(%)	
------						----------	------------		----------------------		------------------
Gender						nominal		Gender			(1=Male;0=Female)			0		
Symptoms 						nominal		Symptoms			(1=Yes;0=No)				10.91
Alcohol						nominal		Alcohol			(1=Yes;0=No)				0
Hepatitis B Surface Antigen		nominal		HBsAg			(1=Yes;0=No)				10.3
Hepatitis B e Antigen			nominal		HBeAg			(1=Yes;0=No)				23.64
Hepatitis B Core Antibody		nominal		HBcAb			(1=Yes;0=No)				14.55
Hepatitis C Virus Antibody		nominal		HCVAb			(1=Yes;0=No)				5.45
Cirrhosis						nominal		Cirrhosis			(1=Yes;0=No)				0
Endemic Countries				nominal		Endemic			(1=Yes;0=No)				23.64
Smoking						nominal		Smoking			(1=Yes;0=No)				24.85
Diabetes						nominal		Diabetes			(1=Yes;0=No)				1.82
Obesity						nominal		Obesity			(1=Yes;0=No)				6.06
Hemochromatosis				nominal		Hemochro			(1=Yes;0=No)				13.94
Arterial Hypertension			nominal		AHT				(1=Yes;0=No)				1.82
Chronic Renal Insufficiency		nominal		CRI				(1=Yes;0=No)				1.21
Human Immunodeficiency Virus	nominal		HIV				(1=Yes;0=No)				8.48
Nonalcoholic Steatohepatitis	nominal		NASH				(1=Yes;0=No)				13.33
Esophageal Varices				nominal		Varices			(1=Yes;0=No)				31.52
Splenomegaly					nominal		Spleno			(1=Yes;0=No)				9.09
Portal Hypertension			nominal		PHT				(1=Yes;0=No)				6.67
Portal Vein Thrombosis			nominal		PVT				(1=Yes;0=No)				1.82
Liver Metastasis				nominal		Metastasis		(1=Yes;0=No)				2.42
Radiological Hallmark			nominal		Hallmark			(1=Yes;0=No)				1.21
Age at diagnosis				integer		Age				20-93					0
Grams of Alcohol per day		continuous	Grams/day			0-500					29.09
Packs of cigarets per year		continuous	Packs/year		0-510					32.12
Performance Status*			ordinal		PS				[0,1,2,3,4,5]				0					
Encephalopathy degree*			ordinal		Encephalopathy	[1,2,3]					0.61
Ascites degree*				ordinal		Ascites			[1,2,3]					1.21
International Normalised Ratio*	continuous	INR				0.84-4.82					2.42
Alpha-Fetoprotein (ng/mL)		continuous	AFP				1.2-1810346				4.85
Haemoglobin (g/dL)				continuous	Hemoglobin		5-18.7					1.82
Mean Corpuscular Volume	 (fl)	continuous	MCV				69.5-119.6				1.82
Leukocytes(G/L)				continuous	Leucocytes		2.2-13000					1.82	
Platelets	(G/L)				continuous	Platelets			1.71-459000				1.82
Albumin (mg/dL)				continuous	Albumin			1.9-4.9					3.64
Total Bilirubin(mg/dL)			continuous	Total Bil			0.3-40.5					3.03
Alanine transaminase (U/L)		continuous	ALT				11-420					2.42
Aspartate transaminase (U/L)	continuous	AST				17-553					1.82
Gamma glutamyl transferase (U/L)	continuous	GGT				23-1575					1.82
Alkaline phosphatase (U/L)		continuous	ALP				1.28-980					1.82
Total Proteins (g/dL)			continuous	TP				3.9-102					6.67
Creatinine (mg/dL)				continuous	Creatinine		0.2-7.6					4.24
Number of Nodules				integer		Nodules			0-5						1.21
Major dimension of nodule (cm)	continuous	Major Dim			1.5-22					12.12
Direct Bilirubin (mg/dL)		continuous	Dir. Bil			0.1-29.3					26.67
Iron	(mcg/dL)					continuous	Iron				0-244					47.88
Oxygen Saturation (%)			continuous	Sat				0-126					48.48
Ferritin (ng/mL)				continuous	Ferritin			0-2230					48.48
Class Attribute				nominal		Class			(1=lives;0=dies)			0

(*) Adicional Info:
PS: [0=Active;1=Restricted;2=Ambulatory;3=Selfcare;4=Disabled;5=Dead]. In this dataset there are only PS from 0 to 4.
Encephalopathy degree: [1=None;2=Grade I/II; 3=Grade III/IV]
Ascites degree: [1=None;2=Mild;3=Moderate to Severe]

More information on HCC dataset’s features can be found in Santos et al. “A new cluster-based oversampling method for improving survival prediction of hepatocellular carcinoma patients”, Journal of biomedical informatics, 58, 49-59, 2015.


8. Missing Attribute Values: Denoted by “?”. Missing percentages for each attribute are specified above.

9. Class Distribution: 
	2 classes:
	63 patients labeled as “dies” (0)
	102 patients labeled as “lives” (1)
