# Supplementary material for: HepDecide: A Standards-Oriented, Explainable Clinical Decision Support Prototype for the Chronic Liver-Disease Spectrum—Design, Guideline-Grounded Reporting, and a Leakage-Controlled Retrospective Evaluation
Source: Healthcare (Basel). 2026 Oct 12;14(20):3397. doi: 10.3390/healthcare14203397 (PMC13704185; doi:10.3390/healthcare14203397)
Supplement: Supplementary file 1 [file healthcare-14-03397-s001.zip › File S4_Training_Evidence.zip!/File S4_LLM_Training_and_Retrieval.pdf]

Supplementary File S4. Language-Model Training and Retrieval

This supplement reports the available training and retrieval records. It does not report a new language-quality evaluation.

Item	Recorded setting
Training data	4104 system–user–assistant records; 1024 distinct contexts, 2549 distinct questions and 4039 distinct answers; no exact whole-record duplicates in the supplied corpus audit.
Model	unsloth/Meta-Llama-3.1-8B-Instruct-bnb-4bit
Adaptation	LoRA rank 64; alpha 128; dropout 0; maximum sequence length 8192
Optimisation	Learning rate 2e-4; batch size 4; gradient accumulation 4; five epochs; seed 3407; 1285 recorded optimisation steps
Validation and stopping	All 4104 records supplied as training data. No validation set or early-stopping criterion defined.
Training loss	Aggregate training loss 0.0498406394; last logged loss 0.022695 at step 1280. The log contains 128 rows.
Source markers	132 records contain 33 distinct source filenames. Lack of a filename marker in 3972 records does not imply absence of context.
Annotation and QC	Annotation authorship, full source mapping and clinical QC procedures are not documented. The current corpus audit does not establish historical clinical review.
Retrieval	BAAI/bge-m3; 800-character chunks; stride 700; overlap 100; cosine similarity; top two; no reranker. PDF chunks of 100 characters or fewer excluded.
Index	3546 chunks reported by the PDF-only notebook step. Repository contains 36 PDFs and three text files; exact saved-index membership unavailable.
Missing evaluation records	Evaluated checkpoint, saved retrieval index, generated answers, metric scripts/results, rater sheets and retrieval latency unavailable.

Prompt and formatting

The system instruction is: “Aşağıdaki tıbbi bağlamı kullanarak kullanıcının sorusunu profesyonel bir doktor gibi cevapla.” In English: “Use the following medical context to answer the user’s question as a professional physician.” The tokenizer chat template formats system, user and assistant messages. The retained notebook supplies the code; it does not show an independent held-out prompt set.

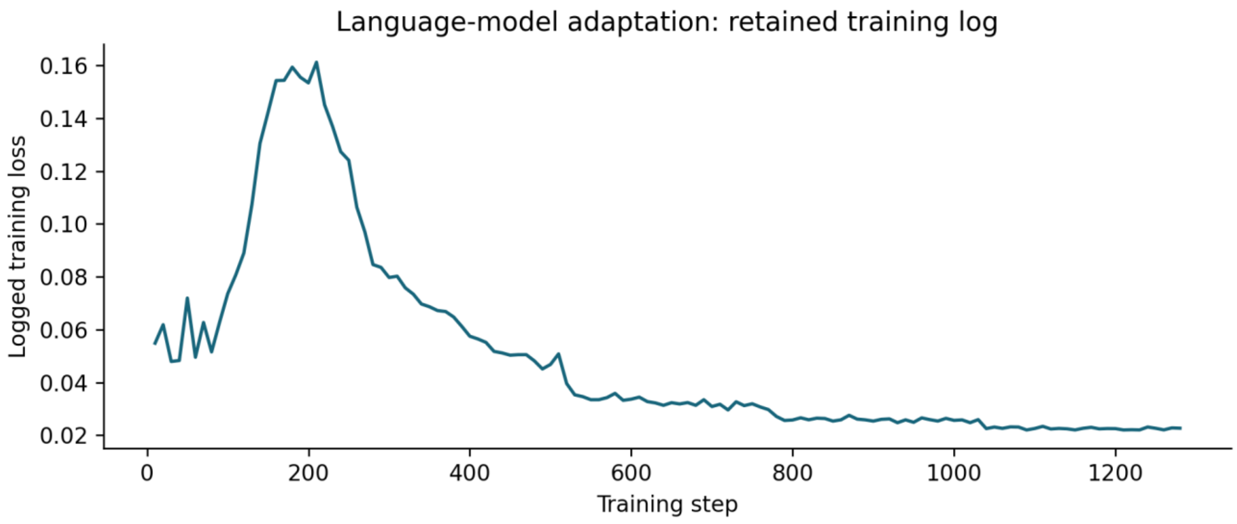

Figure S1. Retained training-loss values by optimisation step. These measurements describe optimisation, not factual accuracy, safety or clinical usefulness.

Relationship to earlier reporting

This rank-64 run cannot be equated with the earlier rank-16 description or a loss near 0.46. The available retrieval configuration also differs from the earlier FAISS/top-six account. Neither can be assigned to the earlier language-performance table without its run records. The notebook records GGUF export and index creation; those binary files were not supplied. A notebook export message is not a substitute for a checkpoint or index.

The evidence files include the loss CSV, source-marker manifest and a training notebook with credential-like strings redacted. The complete corpus is retained in the advisor package; source-text redistribution rights and clinical annotation provenance have not been established.
