# Supplementary material for: HepDecide: A Standards-Oriented, Explainable Clinical Decision Support Prototype for the Chronic Liver-Disease Spectrum—Design, Guideline-Grounded Reporting, and a Leakage-Controlled Retrospective Evaluation
Source: Healthcare (Basel). 2026 Oct 12;14(20):3397. doi: 10.3390/healthcare14203397 (PMC13704185; doi:10.3390/healthcare14203397)
Supplement: Supplementary file 1 [file healthcare-14-03397-s001.zip › File S5_Initial_Training_Records.zip!/File S5_Initial_Experiments.pdf]

Supplementary File S5. Initial Development Experiments

This supplement identifies the evidence supporting the initial results and the limits of their reconstruction. Group B is a separately defined internal analysis.

Module	Initial evaluation	Available evidence and remaining uncertainty
Fatty-liver module	Reported AUC 0.90; archived figure contains 21 evaluation records.	CatBoost and companion files are retained. Model structure is consistent with 81 fitted observations, while the scaler used all 605 source records. Original test identities and the sampled intermediate table are unavailable.
Cirrhosis	AUC 0.9791790600832837; accuracy 0.9268292683; F1 0.925. Test n = 82, 41 per class.	Saved notebook output supports the result. The modelling table had 406 records, 203 per class; train/test 324/82, stratified random_state 42. Both original input tables are no longer available.
HCC survival	Reported AUC 0.92; reconstructed saved-model AUC approximately 0.924.	Reconstruction uses a 41-record partition of the 204-record derivative, stratified seed 42; scaler matches 163 training records. One unresolved AFP value follows the application's zero-input convention. This is a reconstruction, not an original prediction export.
Language layer	Training procedure and loss retained; language-evaluation results not recalculated.	Corpus and notebook document training. Raw answers, rater scores, evaluated checkpoint, retrieval index and metric calculations were not supplied.

Cirrhosis methods and aggregate result

The preparation section reads an 803-record file named birlesik_siroz_temizlenmis.xlsx. Its source composition and patient uniqueness are unresolved. The modelling section reads siroz_dengelenmis.csv, containing 406 balanced records. An intervening export uses a different filename and object, so the saved code does not establish a complete derivation between the two tables. The author-side file search and user confirmation indicate that the two original tables are no longer available.

BMI is filled using single-column KNNImputer and INR and creatinine using their medians before splitting. XGBoost uses raw-scale predictors. Other algorithms fit a scaler on training records after the split. The final search compares 972 XGBoost configurations by three-fold F1 score. The retained result uses colsample_bytree 0.8, gamma 0.5, learning_rate 0.05, max_depth 4, min_child_weight 5, n_estimators 100 and subsample 1.

Actual / predicted	Negative	Positive
Negative	39 (TN)	2 (FP)
Positive	4 (FN)	37 (TP)

Table S1. Counts uniquely implied by the saved aggregate classification report. They were not recovered from patient-level predictions and cannot provide PR, calibration, Brier or decision curves.

Saved-model correspondence

The seven explicit XGBoost settings match the retained binary, but matching settings do not identify a fitted model uniquely. The application's XGBoost branch bypasses its companion scaler and imputer. Their summary statistics therefore do not prove how that XGBoost model was trained.

A conditional tree check also remains unresolved. With binary logistic loss, initial probability 0.5, unit observation weights and no custom margins, the first tree is consistent with 324 observations and 162 positives. Its BMI < 23.12 branch implies 153 observations. In the notebook's displayed BMI summary, 236 values are observed, the first quartile is 28.7 and the mean is approximately 29.918; under the recorded single-column filling procedure this cannot supply 153 values below 23.12. The check depends on the stated weighting and initial-margin assumptions. It indicates an unresolved data, execution-state or model-version difference, not proof of a specific error or misconduct.

What can be concluded

The saved output supports reporting 0.98 as an initial development estimate. It does not establish the identities or probabilities of the original test records. The available model cannot uniquely recover those records. The initial 406-record experiment cannot be labelled as F4 evaluation in the 605-patient cohort, and patient overlap is unknown. No search for a split yielding a desired AUC was performed.

NEW_HCC.ipynb contains a different 204-record, 14-predictor experiment, with SVM AUC approximately 0.688 and XGBoost AUC approximately 0.738. NAFLD_Model.ipynb contains exploration but no recoverable CatBoost fit supporting 0.90. The later server archive adds no original prediction CSV or historical SHAP arrays; its 12 saved model files match the earlier copies by SHA-256. Notebook filenames, hashes, cell evidence and available ROC imagery are supplied in the companion evidence package.
